# Supplementary material for: Genome maps across 26 human populations reveal population-specific patterns of structural variation
Source: Nat Commun. 2019 Mar 4;10:1025. doi: 10.1038/s41467-019-08992-7 (PMC6399254; doi:10.1038/s41467-019-08992-7)
Supplement: Supplementary file 1 — Supplementary Information [file 41467_2019_8992_MOESM1_ESM.pdf]

Supplementary Information

Title of paper: Genome maps across 26 human populations reveal population-specific patterns of structural variation

Corresponding author: Pui-Yan Kwok

Manuscript number: NCOMMS-18-38105-T

Supplementary Methods

1. Analysis of structural variations

1.1. Filtering of SV lists

To produce the final SV lists, we integrated the SVs from the three modules of OMSV. Overlapping SVs of the same type were merged, with the size of the resulting SV estimated by the mean size of the merged SVs, and its span (i.e., the possible occurrence location) set as the union of the spans of the merged SVs.

Since some analyses were sensitive to false positives on the SV list, we performed additional filtering to the high-confidence list by removing the following SVs:

- SVs that overlapped complex regions at which alignments/assemblies could be unreliable. We compiled a list of 55 complex regions (Supplementary Data 3).
- SVs that overlapped unknown sequences on the reference (N-gaps) since these SVs were less reliable. Considering chromosomes 1-22, X and Y of hg38, there were 879 N-gaps, with a total size of 149,690,620 bp.
- SVs that overlapped fragile sites, DNA regions with close nicking sites on opposite strands, which could cause DNA double-strand breakage and wrongly detected as signals of SV.
- SVs that overlapped the pseudo-autosomal regions (PARs) on the X and Y chromosomes, since optical maps from these regions had a high chance of getting aligned incorrectly. In some recent large-scale population genomic studies, PARs were handled by excluding them[3] or masking them on one of the sex chromosomes[4]. We followed the former strategy in this study.
- SVs that overlapped regions with super high density of nicking sites. There were three such genomic regions based on the *in silico* reference map, namely chr10:39687138-39935168, chr12:34820121-37139973, and chr17:22754856-23194891. The average

density of nicking sites in each of these regions was larger than one site per 3 kb, which increased the difficulty of alignment, assembly and subsequently SV detection.

For both the high-confidence list and the full list, we kept only SVs larger than 2 kb to focus on large SVs in this study.

1.2. Definition of populations and super-populations

We adopted the same definitions of populations and super-populations from the 1000 Genomes Project[2,5], which included 26 populations grouped into 5 super- populations, namely AFR (Africans), AMR (Admixed Americans), EAS (East Asians), EUR (Europeans) and SAS (South Asians).

1.3. Validating SVs by 10x Genomics linked sequencing data

We produced 10x Genomics linked sequencing data for 13 samples evenly picked from 5 super-populations. SVs identified from optical mapping were validated by these linked sequencing data if it overlapped an SV called from the sequencing data, or if the flanking sequences supported the SV size.

Specifically, the linked sequencing data from each sample were assembled using Supernova[6]. The resulting contigs were compared to the reference sequence using nucmer from the Mummer package[7]. Segments of changes larger than 1 kb were collected as SVs. An SV identified from optical mapping was considered supported by an SV from linked sequencing if they overlapped and had the same type.

In the flanking sequence analysis (Supplementary Figure S7), for each SV identified from genome mapping, we extracted its flanking sequences on both sides in the reference genome and aligned them to the 10x Genomics contigs. If both flanking sequences could be aligned to the same contig, we compared the distance between them on the contig and the reference to determine whether the 10x Genomics data supported the SV. An SV was considered validated if the distance on the contig was at least 500 bp larger (resp. smaller)

than the reference for an insertion (resp. deletion). If one or both sequences could not be aligned to 10x Genomics contigs, or they could only be aligned to different contigs, we considered the SV not verifiable and did not consider it in the calculation of the supporting rate.

1.4. Analysis of SVs with different sizes in different populations

We performed an analysis of variance (ANOVA), taking each super-population as a group of samples, of the sizes of all *N* detected SVs. We selected SVs with a resulting *p*-value < 0.05/*N* (Bonferroni correction of threshold) and being called in at least 10 samples.

1.5. Analysis of SVs detected in Sudmant et al. (2015)[2] but do not overlap SVs on our high- confidence list

To study the SVs detected in Sudmant et al. (2015) that did not overlap the SVs on our high-confidence list, we classified them into the following categories:

- SVs found on our full list: These SVs could be identified by using less stringent settings of our pipeline.
- Remaining SVs that overlapped one of our filtered regions: Due to the filtering, these SVs could not be detected by our pipeline.
- Remaining SVs having sufficient (≥20) aligned optical maps that overlapped their loci in at least 75% of the samples: Due to the good depth of coverage, a legitimate SV could likely have been detected by our pipeline, and therefore the 1000 Genome calls could be false positives.

1.6. Analysis of specific and common SVs in different super-populations

We studied the ratios of SVs specific to a single super-population and shoes shared by multiple or all super-populations. To handle the issue of having different numbers of samples among different super-populations (e.g. 42 African samples and only 24 American samples), we sub-sampled each super-population to the number of samples of the super-population

with the fewest samples. The sub-sampling procedure was repeated 100 times with different random subsets of samples, and then we took the average of their results.

1.7. Principal component analysis of SV occurrence matrix

We constructed an SV occurrence matrix in which each row was a sample, each column was a high-confidence SV, and each entry was the allele count of an SV in a sample. Rare SVs that appeared in less than 5% of samples were removed. Samples with an SV count three standard deviations or more from the mean were also removed. To further eliminate the effect of SV count, we projected the samples onto the hyperplane orthogonal to the SV count. We performed this by adding the SV count vector as extra columns to the matrix before applying principal component analysis (PCA) and ignored the first principal component (PC). The second and third PCs were then reported as the real first and second PCs of the PCA.

1.8. Phylogenetic analysis of the 26 populations

In the phylogenetic analysis, we again removed rare SVs that appeared in less than 5% of samples from the high-confidence SV list. We then supplied these SVs and their zygosity in each sample to EIGENSOFT[8] to estimate the F_{ST} statistic between each pair of populations. The resulting matrix of F_{ST} values was then used to reconstruct the phylogenetic tree by Neighbor Joining.

1.9. Saturation analysis of SVs

Based on the SVs identified from our samples, we performed a saturation analysis[9,10] to predict the ultimate number of large SVs in human populations. We used the following saturation curve:

$$y = a(1 - e^{-bx^c})$$

where a denotes the ultimate number of SVs, b and c are constants to be determined from

curve fitting, and x is the number of samples. The derivative $y'(x)$ is the number of novel SVs that can be found by including one more sample when there are already x samples.

1.10. Comprehensive analyses of inversions

For the inversions, we identified 338 (out of 380) in the low-complexity regions, 31 of which were also identified by the 1000 Genomes Project. In addition, 72 of 99 inversions in the Sanders callset[11] that can be lifted over to hg38 overlap with our study, suggesting the specificity of our study (Supplementary Table 8-9). Among the remaining 27 inversions not found in our study, 12 of them were found to be close to N-gaps in hg38, making these inversions hard to call confidently, and the other 15 inversions have relatively low allele frequencies in the Sanders callset (average 0.25 for these inversions as compared to average 0.36 for the others) and a lower proportion of them can be found in the DGV database (33% for these inversions as compared to 81% for the others). These findings suggest them some of these inversions are rare or false positives.

We also explored possible population structures based on the complex structural variations. From the PCA based on the inversions, the African samples were separated from the others based on the first two PCs and showed a clear cluster in the heat map (Supplementary Figure S26). However, the other four super populations were not well separated, suggesting that few novel inversions have emerged in those populations. We also found some inversions identified from at least three samples to be specific to a single super-population, including 7 identified in African samples, 4 in East Asian samples and 1 in European samples.

2. Y chromosome analysis

2.1. Y chromosome assembly

Consensus maps generated from the Bionano IrysSolve *de novo* assembly pipeline were

aligned to the *in-silico* labeled reference map. Coordinates of the maps aligning to the Y chromosome were extracted and the overall sample coverage was computed along the chromosome. The Illumina callable regions (Supplementary Figure S22) were obtained from Poznik, et al.[12] and the genome coordinates were converted to hg38 using liftOver. Coordinates corresponding to the segmental duplications were downloaded using the UCSC Table Browser[13] and repeats with greater than 95% match fraction were used for plotting.

2.2. Identifying SV candidates in the Y chromosome

A list of SV candidates was manually curated using the top ten samples with the longest molecule N50s and the highest genome-wide coverages. These samples have long assembled contigs that can be used to confidently locate non-reference alleles on the Y chromosome for downstream analysis. SV candidates from these ten samples were initially verified using single molecules. Insertions, deletions, and inversions that were located inside segmental duplications were tolerated as long as the SV candidates were within 150 kb to a unique anchor on at least one end. After determining the genomic locus of each SV, we genotyped all samples using one of the following two strategies depending on the SV type.

2.3. Insertion, deletion, and inversion analysis

To detect insertions, deletions, and inversions among all male samples, an *in silico* labeled representation of each haplotype was created in CMAP format using custom scripts that combined the flanking areas of the reference chromosome with areas of representative assembled contigs observed in our samples. For all haplotypes at a given locus, their CMAP representations were kept as consistent between one another as possible, i.e. containing the same flanking areas. For each locus, single molecules from each sample were used to determine which haplotype(s) the sample contained, as follows:

1) Using outputs from the standard Bionano *de novo* genome assembly pipeline, all of the single molecules that aligned to the local area of the reference genome were obtained.
2) The local molecules were re-aligned to a CMAP file containing each haplotype in the locus using OMBlastMapper from OMTools[14] version 1.4a with the following parameters: --writeunmap false --alignmentjoinmode 1 --filtermode 1 --trimmode 1 --minconf 0 --minjoinscore 0 --maxalignitem 2.
3) The resulting alignment files were post-processed. First, the top two alignments for each molecule were compared to one another; if they received the same confidence or score, they were discarded. Otherwise, the best hit for each molecule was evaluated to see whether its alignment spanned the entire "critical region" (CR). These regions were defined as those that were unique to the target haplotype when compared to other haplotypes at the same locus, as well as being anchored in the flanking area(s) by at least four labels or 40 kb, whichever was longer. For inversions inside palindromes, two CRs are defined, one on each end. Molecules would only be required to span one CR. Due to the inherent noise of single molecules, indels were permitted in the alignment overlapping the CR as long as the size change due to the indel was smaller than 50 kb.
4) Molecule alignments were manually verified if a haplotype for a given sample was supported by only one or two molecules, or if less than 70% of the molecules supported the chosen haplotype. For these flagged cases, the alignment between the molecules and haplotypes were manually inspected in OMTools OMView.

2.4. Copy Number Variation analysis

1) For each sample, we identified and extracted local molecules that aligned to the CNV candidate locus based on the initial outputs from the standard Bionano *de novo* genome assembly pipeline.
2) We made a CMAP of only the unique region immediately adjacent to but not including the CNV. We then realigned all Y chromosome molecules to this CMAP with OMBlastMapper from the OMTools package to gather as many informative molecules as possible. The non-redundant molecules were appended to the initial list of aligned molecules from step 1.
3) All molecules from the list were aligned to the corresponding CMAP containing the CNV using OMBlastMapper with the following parameters: –alignmentjoinmode 1 –filtermode 1 –maxaligneditem 1 –trimmode 1 –overlapmergemode 0. Alignment output was filtered to keep molecules that were anchored to both ends of the CNV, and at least one anchor had to be unique.
4) Filtered alignment output was passed to the SVDetection program of the OMTools package. A minimum support of 1 molecule was used to identify the overall size change between the flanking anchors. If more than one size change was reported, the result with the highest number of molecule support was used for the copy number calculations. Since the repeat unit is 23 kb for both CNVs, we divided the overall size change by this number to obtain the final copy number.
5) We manually determined the copy number if the division ended within the range of 0.3 – 0.7. Otherwise, the numbers were rounded to the closest integers. If no molecule could anchor to both ends of a CNV site, the particular sample would be removed from downstream analysis.

3. Identification of novel genome content not found in the hg38 reference

Non-aligned contigs were gathered from all 154 genomes by comparing contig IDs that appeared in the final hg38-aligned XMAP file to the total assembled contigs. Contigs not appearing in the XMAP file were denoted as non-aligned contigs and *de novo* assembled using the Bionano IrysSolve assembly pipeline with default settings (pipeline version 4618). Assembly resulted in 42 contigs for a total summed length of 16 Mb. Contigs not participating in this assembly were all-against-all aligned with Bionano RefAligner using default parameters. Alignments were filtered by confidence score of 1e-11. The alignment output XMAP file was loaded into a python pandas dataframe (python version 3.6.0, pandas version 0.22.0) and grouped by query contig ID (column 2). All collapsed groups were intersected by shared contig IDs. Groups with one unique ID - in other words, groups with no additional alignment to other contigs - were removed from consideration. The remaining unique groups totaled ~46 Mb in summed length.

4. N-gap closing

4.1. Genome map data

N-gaps in the hg38 reference that were fully closed in our dataset were identified as follows. For every sample with genome map data, beginning with an alignment of the assembled genome map contigs to the reference produced by the *de novo* assembly pipeline (with parameters -res 2.9 -FP 0.6 -FN 0.06 -sf 0.20 -sd 0.0 -sr 0.01 -extend 1 -outlier 0.0001 -endoutlier 0.001 -PVendoutlier -deltaX 12 -deltaY 12 -xmapchim 12 5000 -hashgen 5 7 2.4 1.5 0.05 5.0 1 1 3 -hash -hashdelta 50 10 -hashMultiMatch 100 10 -insertThreads 4 -nosplit 2 -biaswt 0 -T 1e-12 -S -1000 -indel -PVres 2 -rres 0.9 -MaxSE 0.5 -MinSF 0.15 -HSDrange 1.0 -outlierBC -outlierLambda 20.0 -outlierType1 0 -xmapUnique 12 -AlignRes 2. -outlierExtend 12 24 -Kmax 12 -resEstimate -f -MultiMatches 5 -MultiMatchesDelta 50.0), for each contig,

the chromosome with the highest alignment score was identified and all alignments for that contig to other chromosomes were discarded. Using Bedtools[15] and custom Python scripts, reference N-gap regions were identified that were either completely spanned by a single alignment, or that were flanked on both sides by non-overlapping, adjacent, same-strand alignments from the same contig. To calculate gap size, the two labels flanking a given gap were identified, and gaps with alignments that did not involve those two labels were filtered out. To avoid ambiguous placement, contigs were filtered out if either of their gap-flanking labels were involved in more than one alignment. Contigs were also filtered if either of the gap-flanking labels were the last label of an alignment, since a single label was not sufficient to confidently anchor the alignment across the gap. Observed gap size was calculated as:

$$label\_distance_{contig} - label\_distance_{reference} + reference\_gap\_size$$

where label_distance is the distance between the two labels flanking the gap area in either the assembled contig or the reference.

4.2. 10x Genomics linked sequencing data

For every sample with 10x Genomics linked read data, samples were assembled using Supernova version 1.1 with default parameters, and both pseudohaplotypes were output. Each pseudohaplotype was aligned to the hg38 reference genome using nucmer from the MUMmer package[7] with settings -maxmatch -l 100 -c 500, filtered using delta-filter -q, and further filtered and converted to a more parsable format using show-coords -Tcrodl -I 90. Using a combination of Bedtools commands and custom Python scripts, scaffolds were identified that aligned to at least 50% of both 5 kb regions flanking a given reference gap. Ambiguous alignments, e.g. cases where multiple areas of the scaffold aligned to the same area flanking a reference gap, were filtered out. Alignments to both flanking regions were required to be on the same strand of the scaffold and to be in orientation-appropriate order,

i.e. for plus-strand alignments, the scaffold region that aligned to the upstream reference region must be upstream of the scaffold region that aligned to the downstream reference region, although some overlap was allowed, as in the case of deletions involving repetitive content around the gap area. For cases where two separate alignments corresponded to the two flanking regions, the 10xG gap length was calculated as follows, for plus-strand alignments on the scaffold:

$$(D_{qs} - U_{qe}) - (D_{rs} – U_{re}) + (G_{re} – G_{rs})$$

or, for minus-strand alignments on the scaffold:

$$(U_{qe} - D_{qs}) - (D_{rs} – U_{re}) + (G_{re} – G_{rs})$$

where U and D are the upstream and downstream alignments with respect to the gap on the reference, respectively, q indicates a scaffold-based coordinate, r indicates a reference-based coordinate, and s and e indicate the start and end, i.e. the smallest and largest coordinates with respect to the reference, of the associated alignments.

For single alignments that spanned the entire gap on both sides, in order to precisely define the coordinates on the scaffold that corresponded to the gap region, the entire scaffold on which the alignment was found was re-aligned to the two 5 kb regions flanking the gap on the reference genome, using lastz[16] with the parameters --seed=match15 --exact=50 --nogapped --notransition --gfextend --chain --filter=nmatch:100 --filter=identity:95. Gap lengths were calculated as above, where G_{re}=0 and G_{rs}=5000.

For all alignments, in cases where the gap length was positive, the scaffold sequence that corresponded to the reference gap region was extracted and processed with RepeatMasker[17] with parameters -species human -xm.

5. Sequence content of SVs

5.1. SV calls using linked-read sequence data

Sequence-based SV calling was done for 13 samples using two approaches: A) Linked reads were aligned to hg38 for phasing and variant calling using the 10x Genomics Long Ranger pipeline (v2.1, WGS analysis, using FreeBayes). The SV calls produced by this pipeline included mid-scale deletions (50 bp - 30 kb) and large-scale SVs (≥ 30 kb). B) *De novo* assemblies produced using the 10xG Supernova software[6] were aligned to hg38 using nucmer (MUMmer v3.23, -maxmatch -l 100 -c 500) of the Mummer package[7]. Assemblies were initially generated using Supernova v1.1. In the course of this study, Supernova v2.0 was released, and we used it to generate new assemblies for these samples. As we aimed to use this sequence data to analyze as many of the optical mapping SVs as possible, we chose to use both set of assemblies for this particular analysis. For each of the 13 samples, nucmer alignment delta files of both of the *de novo* pseudohaplotypes (outputs designated by Supernova as 2.1 and 2.2) were input to Assemblytics[18] for SV calling, using minimum alignment lengths of 5 kb, 10 kb, 50 kb, 100 kb, 250 kb, and 1 Mb. Scaffolds were used rather than contigs because gaps in Supernova assemblies consist of a sequence of Ns roughly approximating the size of the gap.

5.2. Filtering the list of SVs found by optical mapping

10X Genomics compiled a ‘blacklist’ for SV calls, representing a set of regions in which SV calls are more likely to represent false positive or otherwise inaccurate SV calls. The regions in the suggested blacklist were derived from: 1) the UCSC browser gap track, including short arm gaps, heterochromatin gaps, telomere gaps, gaps between contigs in scaffolds and gaps between scaffolds in chromosome assemblies; 2) segmental duplications of >= 1Kb and

>=90% sequence identity between copies; and 3) regions with new sequences introduced in GRCh38 (hg19 diff track, UCSC browser)[13].

SVs that were identified by optical mapping but were within 20 kb of a region on the blacklist were filtered out and not included in downstream analysis.

5.3. Locating SV breakpoints and their associated sequences

Deletion breakpoints identified by optical mapping were compared to deletion breakpoints identified in the same sample by Long Ranger and Assemblytics (including deletions, repeat contractions, and tandem contractions). Insertion breakpoints identified by optical mapping were compared to large duplications identified in the same sample by Long Ranger and to insertions, repeat expansions, and tandem expansions identified in the same sample by Assemblytics. Comparison and interval intersection was done using the bedtools[15] package. Matching records were retained when at least a third of the SV size was supported by the complementary method. Up to a 20 kb difference between breakpoint positions was permitted in order to account for the lower resolution and potential local misalignments of the optical maps. Next, the SV call lists of the 13 samples were merged into a unified list such that each of the SVs that were found by optical mapping had only one entry, annotated by the best matching sequence-based SV call. Ranking of matched sequence-based SVs was based on SV size and type as classified by Long Ranger and Assemblytics, where deletions and insertions were ranked higher than tandem contractions and expansions, which were themselves ranked higher than repeat contractions and expansions.

After optical map SVs were annotated with more accurate breakpoints based on the sequence-based SV calls, the corresponding sequence was extracted from either the reference or *de novo* assemblies, in the case of deletions or insertions, respectively. If the sequence contained mainly Ns, it was recorded as ‘N-gap’. To determine the repetitive

content of the SVs, we used RepeatMasker[17] v4.0.7 (--species human --xsmall). Based on the results, SVs were assigned to content classes (SINEs, LINEs, LTR, DNA, satellites, simple, unclassified, or low) when at least 50% of the sequence was of the same repeat class. SVs were classified as 'combined' when the repeat content was > 50% but no single class contributed 50% or more to the sequence content. SVs with repeat content < 50% were classified as 'none' unless they were classified as tandem repeats by Assemblytics or as pseudogenes in downstream analysis (see below).

Sequences flanking the breakpoints of SVs were also processed to identify the involvement of repetitive elements. The regions 500 bp before and after each SV were analyzed using RepeatMasker. The flanking regions were assigned to the main repetitive classes listed above, with a minimum requirement of 100 bp per repeat type.

5.4. Identification of pseudogenes involved in SVs

To identify SVs that originated from retrotransposition activity but could not be classified by RepeatMasker, we also looked for processed pseudogenes[19]. We used the Pseudogenes annotation of the Retroposed Genes V9 UCSC browser track[13] to identify pseudogene sequences from the reference that were deleted in the 13 samples. To identify insertions that were likely a result of a processed pseudogene retrotransposition event, we created a set of 40 bp chimeric sequences for every gene in the genome, composed of the last 20 bp of exon n and the first 20 bp of exon n+1 of the same gene. We aligned these sequences to the *de novo* assemblies using Bowtie 2[20] with a minimum alignment threshold of 17 matches out of 20 bp. To filter out hits from pseudogenes present in the reference genome, lastz[16] was used to perform local alignment of the candidate pseudogene and its flanking sequence (spanning 2x the size of the pseudogene) to the reference. The EMBOSS[21] stretcher global alignment tool was used to align all candidate pseudogenes to their corresponding cDNA sequences to

determine the involved exons and removed introns, and candidates with low quality alignments (match sites <200 or similarity <90%) or without clear loss of introns (minimum of 90% of intron lost) were discarded. For additional filtering, anchor sequences (typically 30 kb upstream and 30 kb downstream, or 100 kb each when 30 kb was insufficient) flanking each of the candidate pseudogenes were aligned to the reference genome using BLASTn[22]. Any alignments larger than 500 bp were recorded and adjacent alignments of both anchors (distance <10 kb + size of pseudogene) were combined into one alignment. Candidate pseudogenes were filtered out if they were found to be present in the reference genome. Finally, EMBOSS stretcher was used to perform global alignment of candidate sequences and their flanking anchor sequences to the reference in order to verify the insertion location. The insertion location and size were then compared to insertions found by optical mapping in order to classify the optical map insertion calls as retrotransposition events.

Supplementary Figures

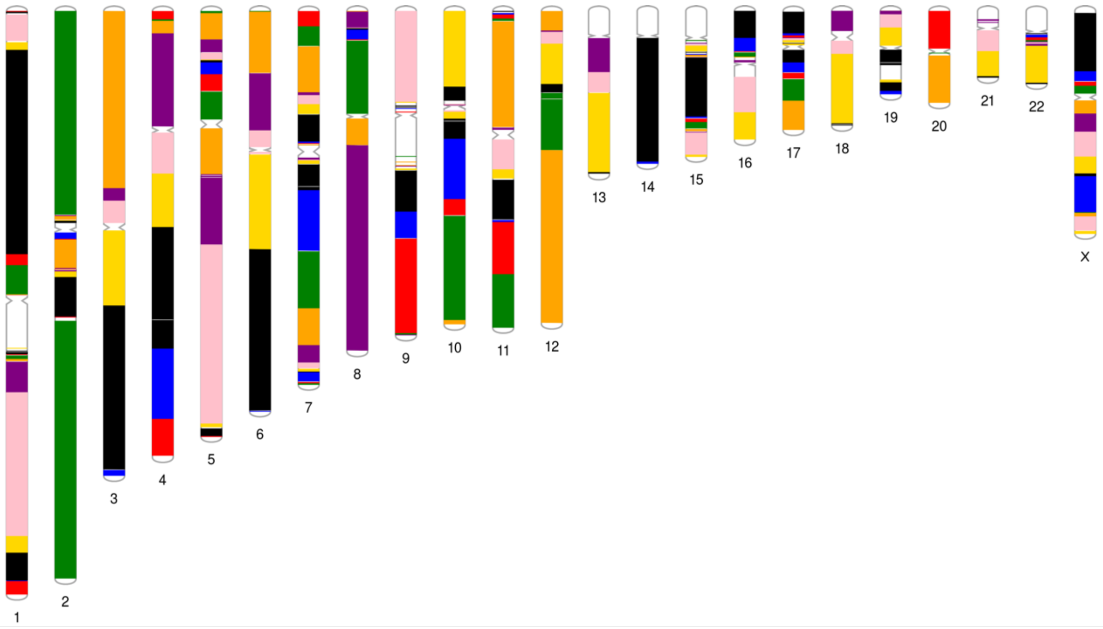

Supplementary Figure 1. Hybrid assembly of NA19440. Assembly based on genome maps and supernova scaffolds as aligned to hg38. Each colored block in the ideogram represents one assembled scaffold. Many of the scaffolds are long, with N50 of 25-35 Mb. Ideogram generated using PhenoGram[1].

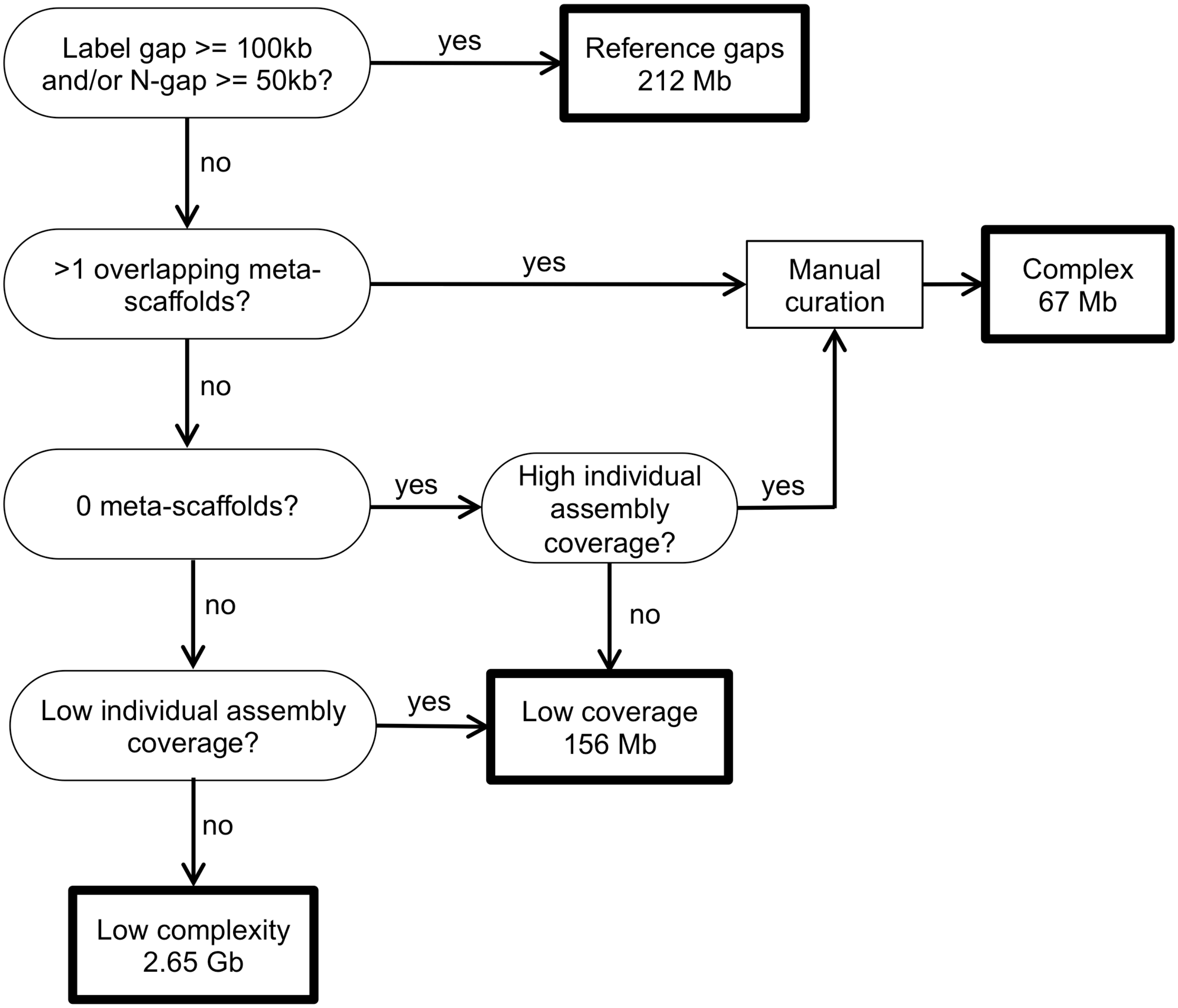

Supplementary Figure 2. Flowchart describing the process for classifying the genome into distinct categories and defining complex regions.

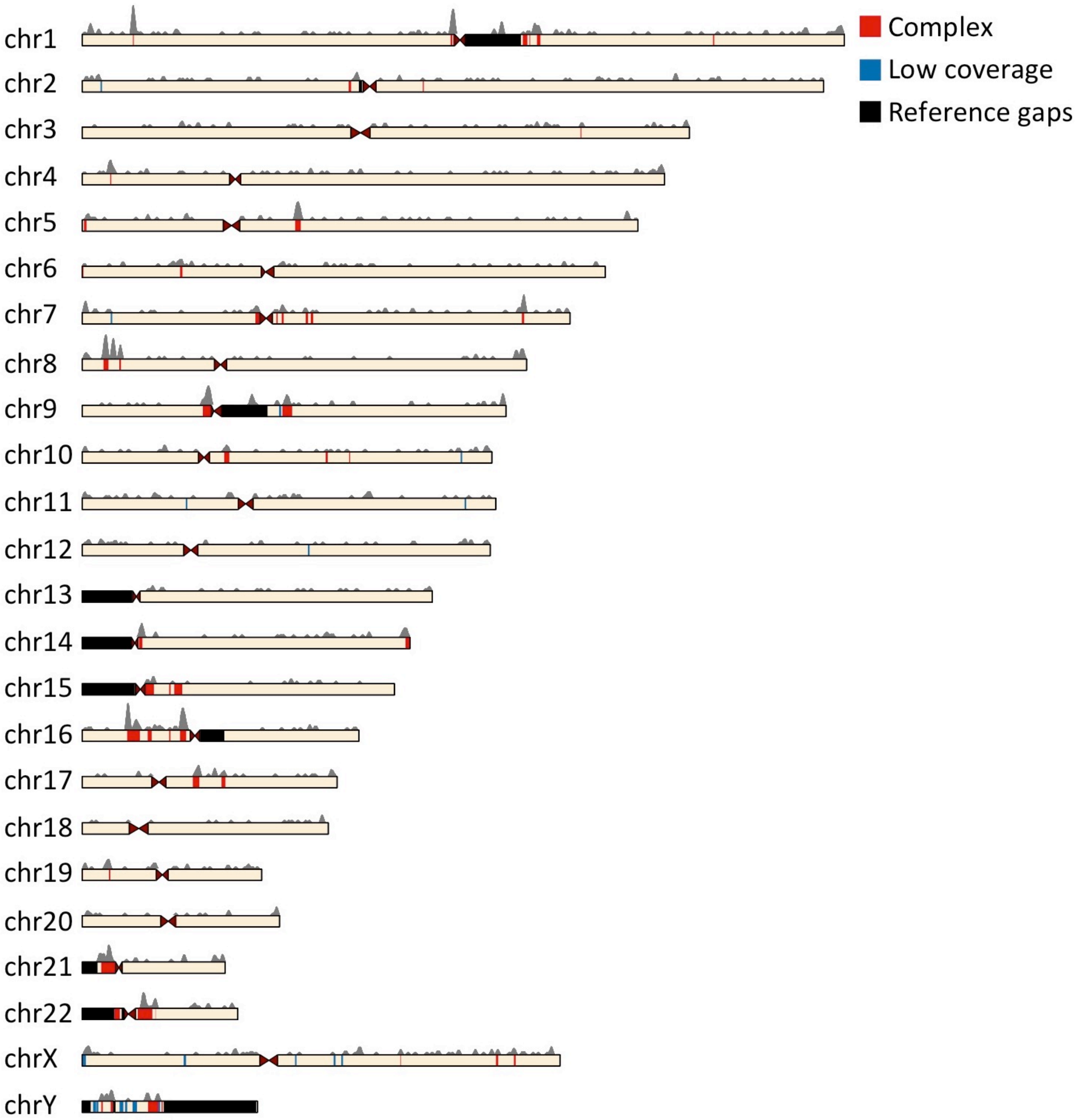

Supplementary Figure 3. Ideogram showing the locations of structural variants detected in the consensus assembly. The grey histogram above the chromosomes depicts the number of SVs detected in the consensus assembly using a sliding window of 1 Mb with a 10 kb step size. Chromosome fill shows the different regions classified in the genome, as in Figure 1. Red, structurally complex regions; blue, low individual assembly coverage; black, regions with long sequence- or nick-based gaps in the reference. For display purposes, both low coverage and gap regions were only displayed if they were longer than 500 kb.

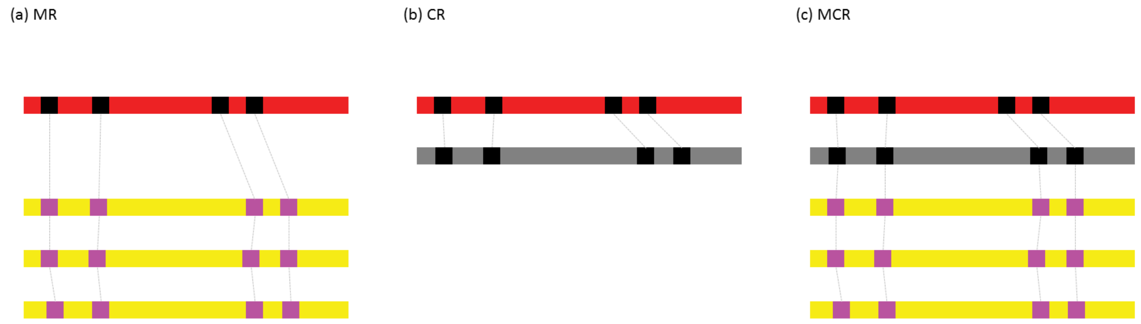

Supplementary Figure 4. The three strategies used in identifying SVs. (a) Direct alignment of individual optical maps to the reference, (b) alignment of contigs assembled from individual optical maps to the reference, and (c) indirect alignment of individual optical maps to the reference by combining their alignments to the contig and the alignment of the contig to the reference. The red, yellow and gray horizontal bars represent the reference, individual optical maps and a contig, respectively. The small black and pink boxes represent nicking sites, and the gray dotted lines represent their alignments.

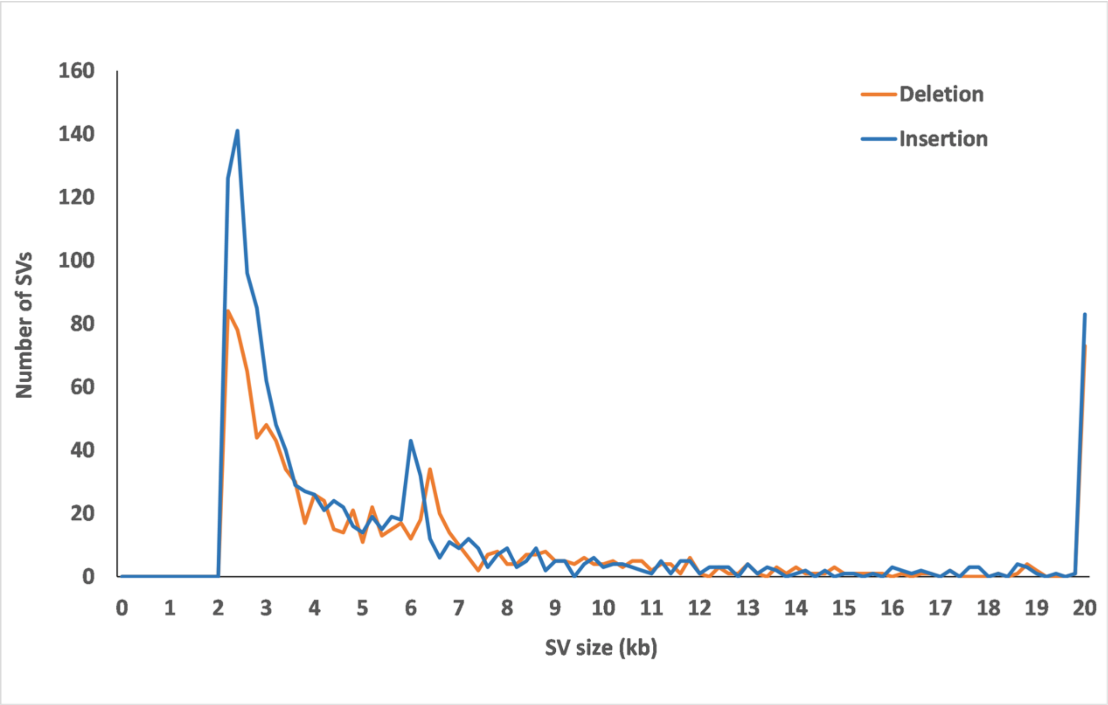

Supplementary Figure 5. Size distribution of structural variations found. The size distribution of deletions (orange) and insertions (blue) with minor allele frequency of at list 0.1 demonstrates a peak at ~6-7 kb, corresponding to LINE1 elements. The peak at around 2 kb is the result of the lowest size threshold used for SV calling (2 kb).

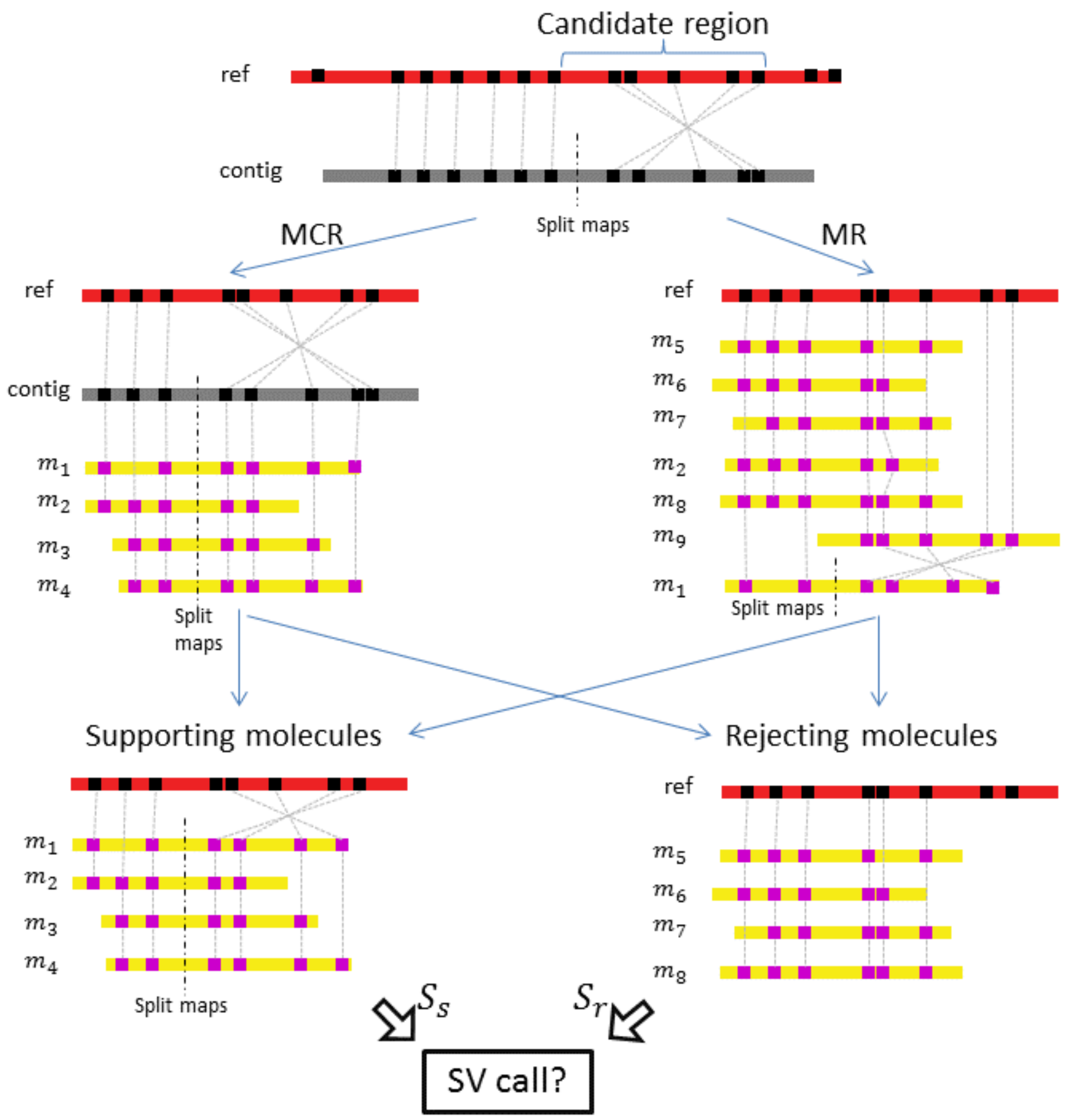

Supplementary Figure 6. The procedure for detecting complex SVs. Candidate complex SVs are identified from split-alignments. Optical maps that support each SV candidate and those that do not support it are collected to determine whether the SVs should be called or not. The red, yellow and gray horizontal bars represent the reference, individual molecules and a contig, respectively. The small black and pink boxes represent nicking sites, the gray dotted lines represent their alignments, and the black dashed-dotted lines indicate the rough location of an SV break point.

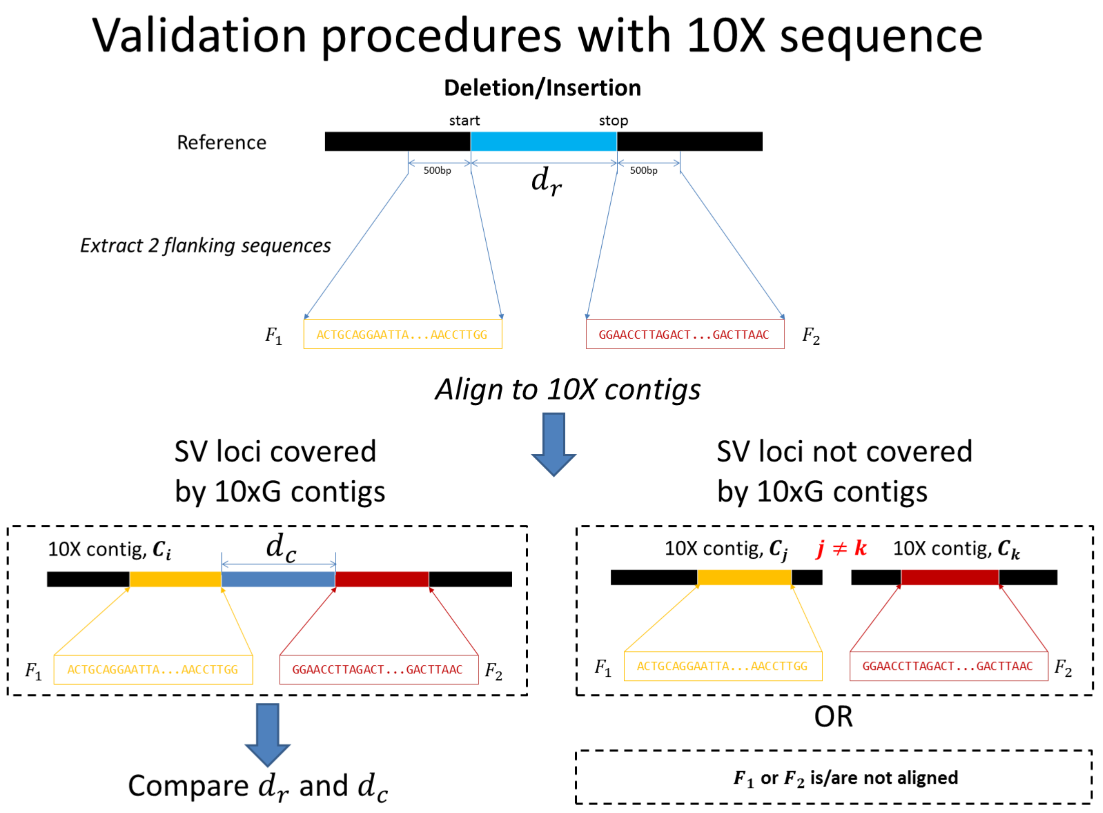

Supplementary Figure 7. Flanking sequence analysis based on 10xG data. For each indel, flanking sequences on the reference were extracted and aligned to the contigs assembled from linked sequencing data. If both sequences were aligned to the same contig, the distance between them would be compared with the distance on the reference to evaluate whether the indel is confirmed by the 10xG data. The indel loci would not covered by 10xG contigs if the two flanking sequences were not aligned to the same contig.

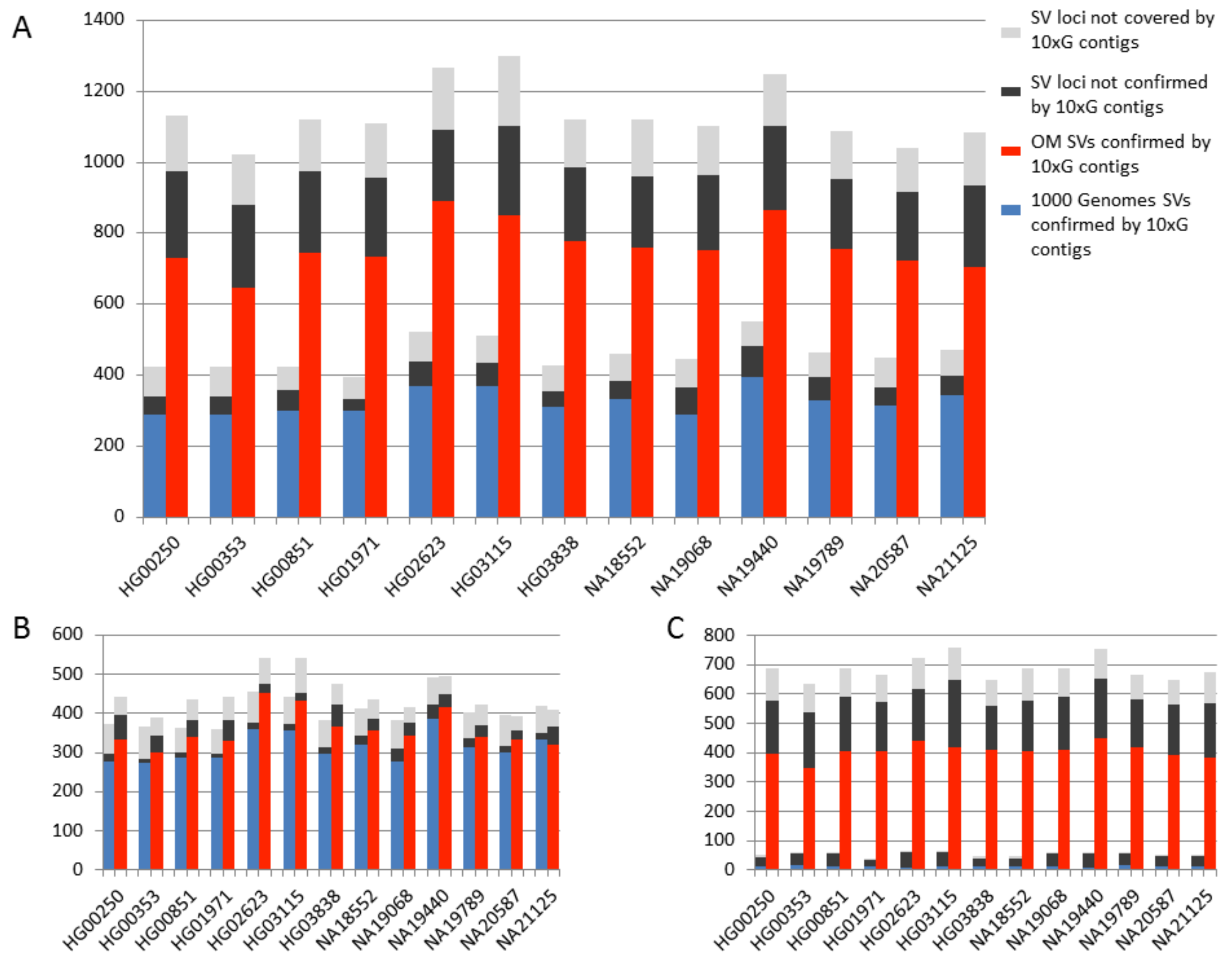

Supplementary Figure 8. Comparisons between SVs. (A) all filtered high-confidence indels, (B) filtered high-confidence deletions, and (C) filtered high-confidence insertions identified by genome mapping in this study and the ones identified by sequencing in Sudmant et al. (2015)[2] (1000 Genomes) based on the 13 samples having 10xG data in this study. Each bar shows the accumulated number of indels identified in the two studies that are directly supported (red and blue) by 10x Genomics linked sequencing data, not confirmed by 10xG contigs (light grey), or having the loci not covered by 10xG contigs (dark grey). Within each bar group, the first bar corresponds to the SVs identified in this study and the second bar corresponds to the SVs identified by Sudmant et al. (2015).

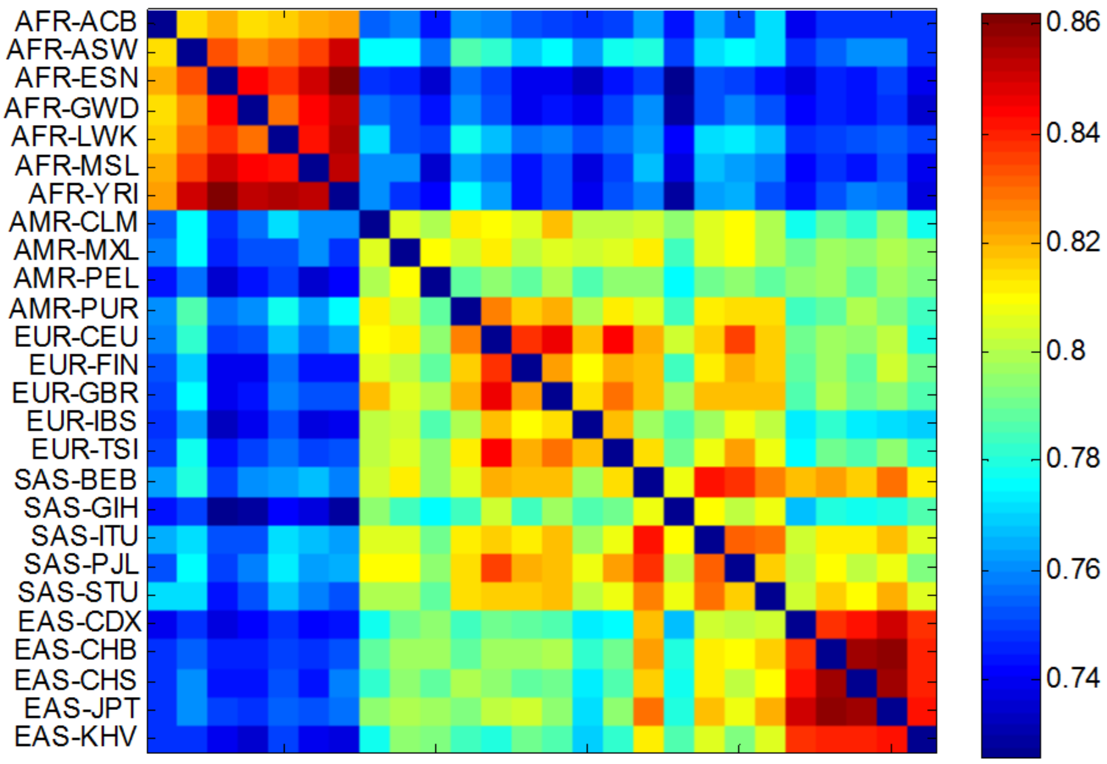

Supplementary Figure 9. Population structure of SVs. Each row and each column represents one population, and the color of each entry represents the similarity between the corresponding populations based on the filtered high-confidence indels. Super-population abbreviations: AFR - Africans; AMR - Americans; EAS - East Asians; EUR - Europeans; SAS - South Asians.

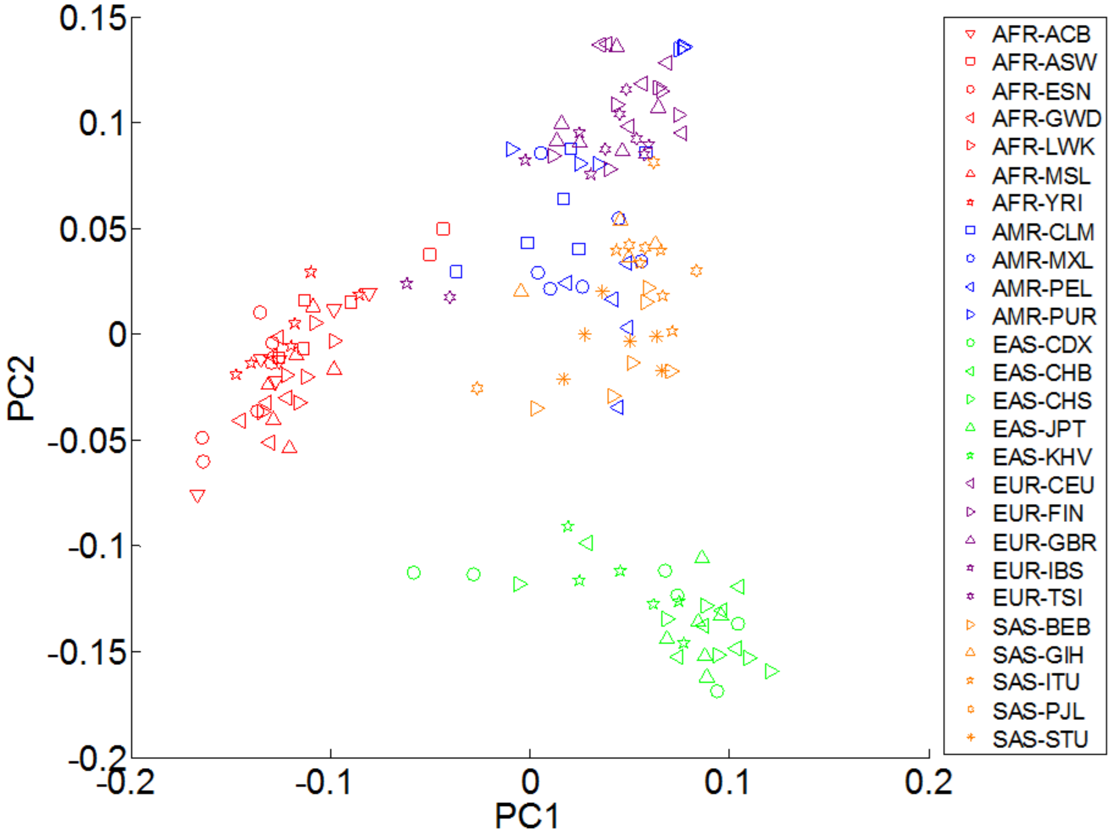

Supplementary Figure 10. Principal component analysis based on the indels occurrence matrix of the filtered high-confidence indels. Super-population abbreviations: AFR - Africans; AMR - Americans; EAS - East Asians; EUR - Europeans; SAS - South Asians.

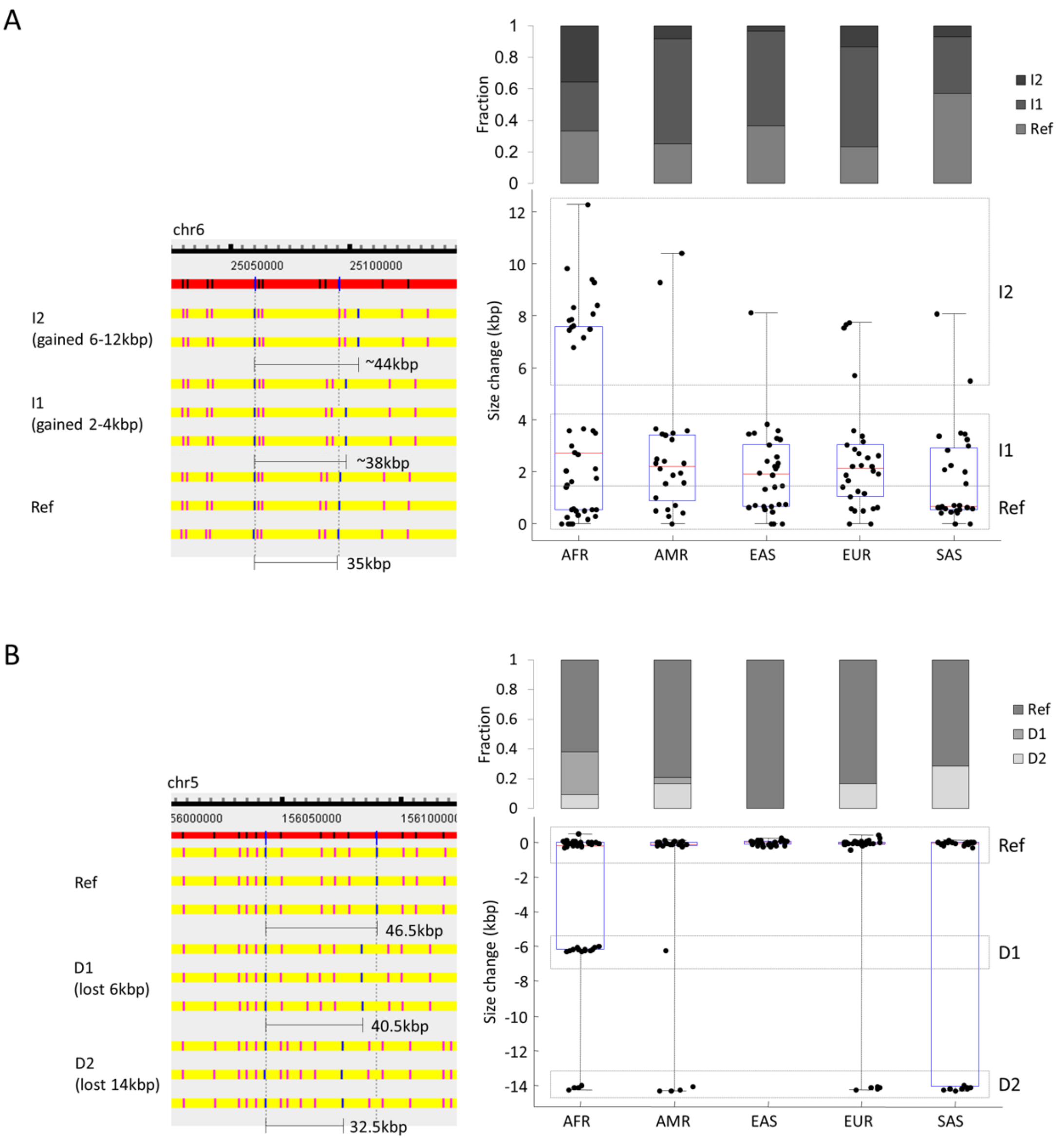

Supplementary Figure 11. Population specificity of the size of indels. The two panels show examples of (A) an insertion, (B) a deletion. In each case, on the left are the alignments of some contigs from 154 samples to the reference map, and on the right is a box plot that indicates the SV size change with respect to the reference allele of the samples in different populations, with the outside vertical bars showing the frequencies of the alleles in each super-population. In the contig alignments, red horizontal bars show the reference, with the nicking sites marked in black vertical lines. Each yellow horizontal bar represents a contig, with the two aligned nicking site labels defining the SVs in blue, other aligned labels in pink, and unaligned labels in black. "I1" and "I2" are different insertion alleles, "D1" and "D2" are different deletion alleles, and "WT" is the wild type (reference).

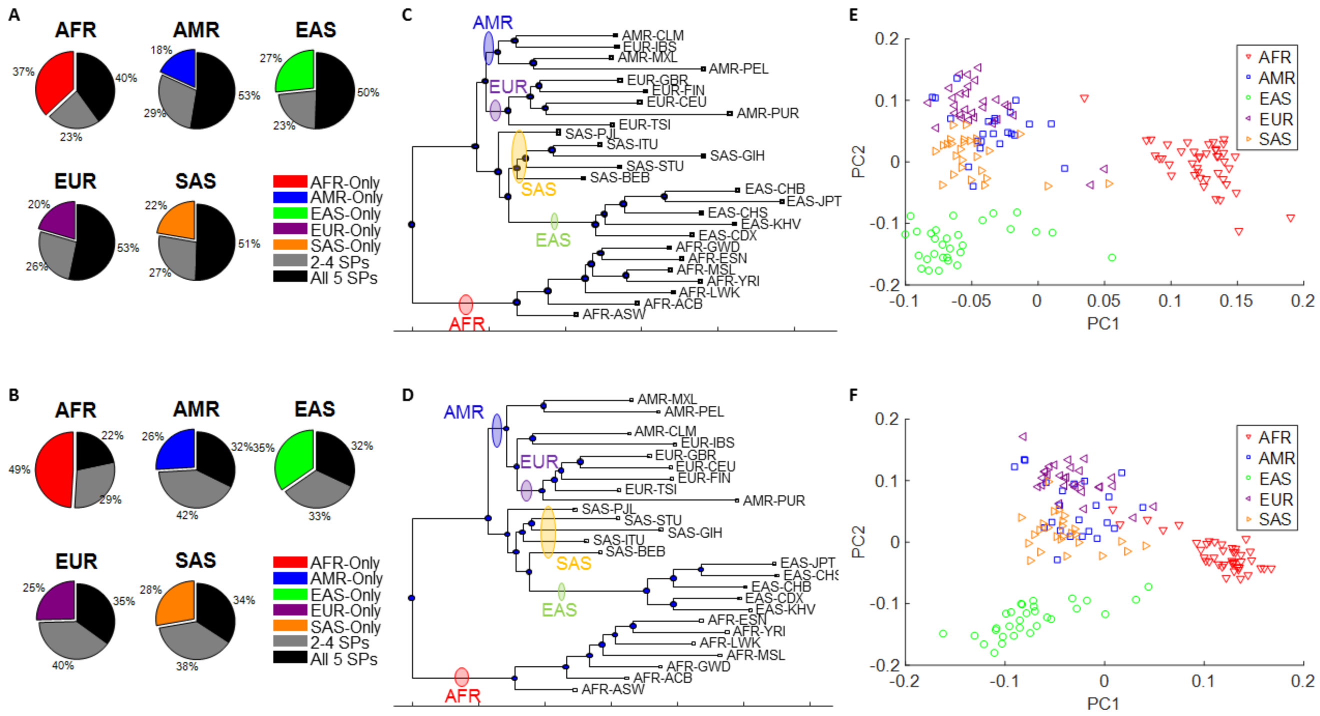

Supplementary Figure 12. Population structure of large insertions and large deletions at three different levels. A,B) Super-population level: The average ratio of insertions (A)/deletions (B) identified from samples in each super-population that are specific to that super-population, shared with some other super-populations but not all, or shared with all other super-populations. Random sub-sampling has been applied to balance the sizes of super-populations. The reported values are the average of 100 random sub-samples. C,D) Population level: A phylogenetic tree constructed based on the insertion (C)/deletion (D) occurrence matrix. E,F) Single-sample level: The first two principal components of the insertion (E)/deletion (F) occurrence matrix based on super-population groups. Super-population abbreviations: AFR - Africans; AMR - Americans; EAS - East Asians; EUR - Europeans; SAS - South Asians.

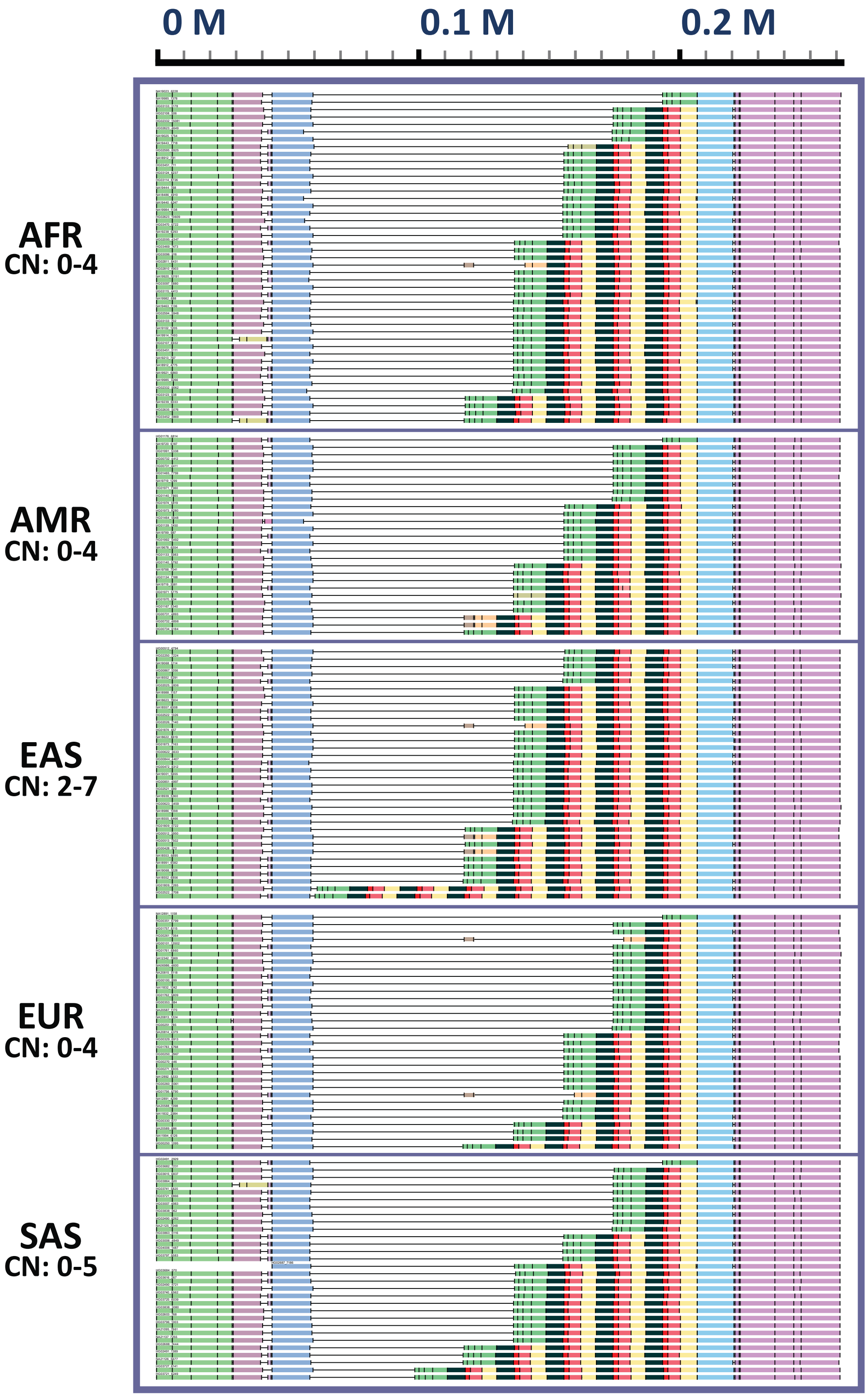

Supplementary Figure 13. Copy number variation in the pepsinogen A gene. Multiple alignment was performed using all assembled OM contigs across the 26 populations at the pepsinogen A gene region. The variable region is flanked by consensus regions on both ends, and is colored to show the variable numbers of repetitive unit among individuals within each super-population.

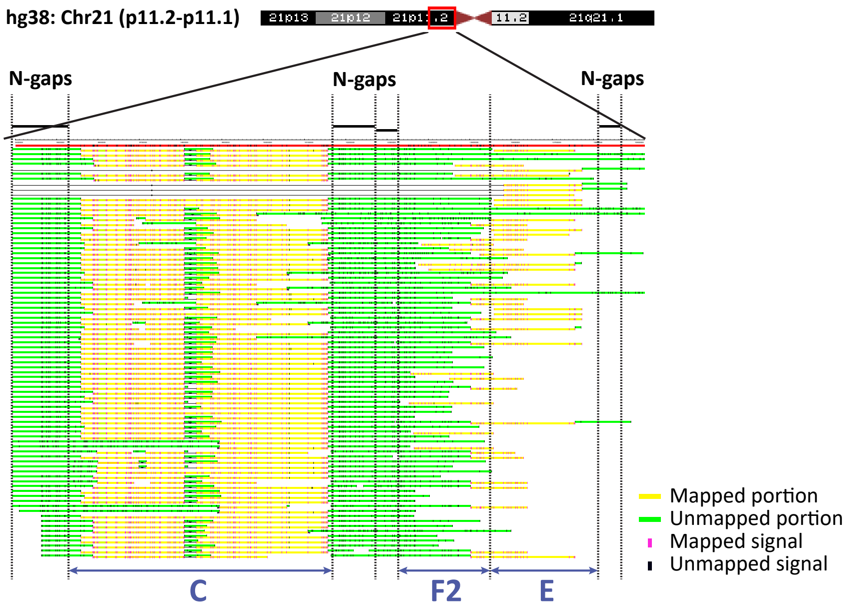

Supplementary Figure 14. Reference alignment of assembled genome map contigs along 21p11.2 of hg38 showing extensive unaligned regions. Assembled contigs across the 26 populations with regions highlighted in yellow and green representing aligned and unaligned regions, respectively. Similarly, consensus labels in pink and black along the contigs represent aligned and unaligned signals, respectively. Blue arrows below indicate the previously reported sub-regions: C, F2, and E, with extensive unaligned regions (in green) interspersed, suggesting a highly complex population structure of this region.

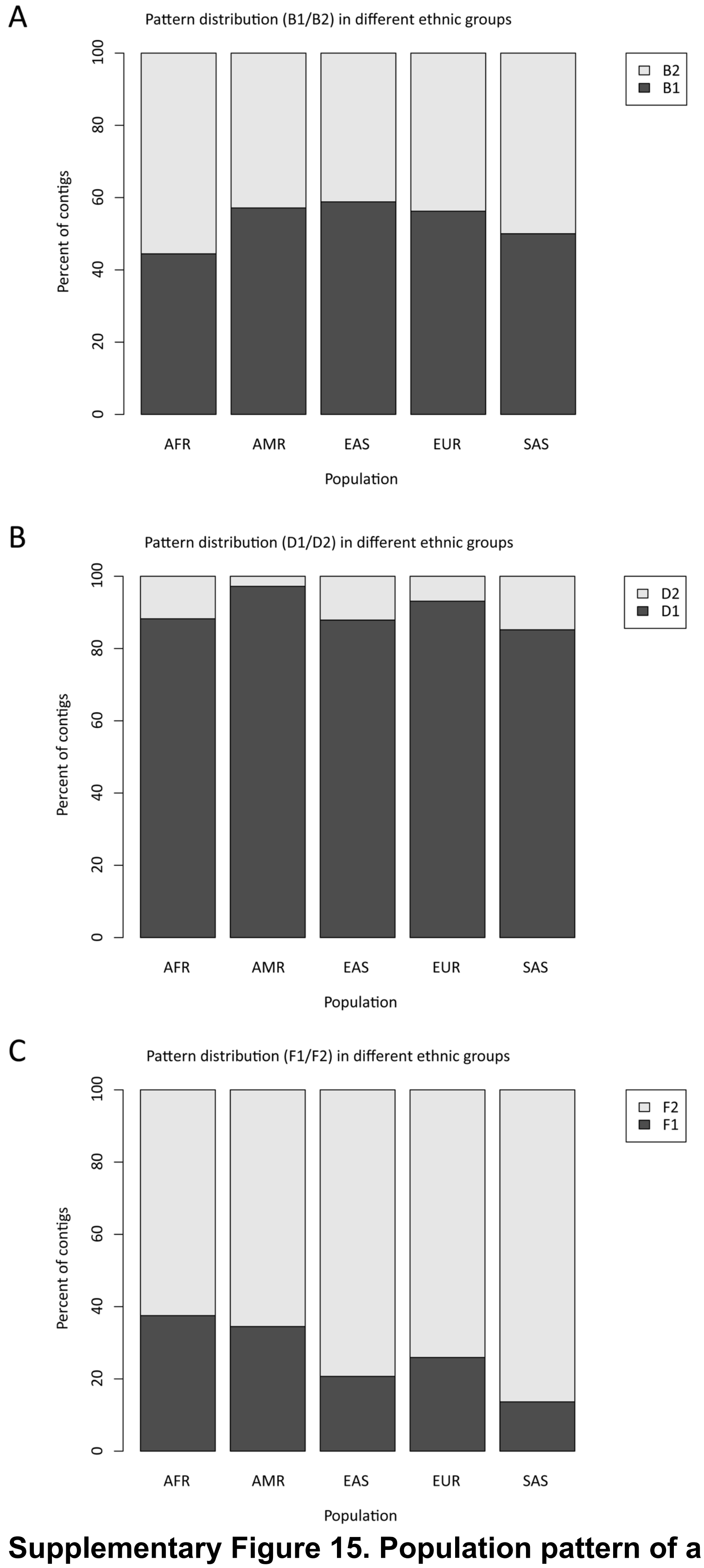

Supplementary Figure 15. Population pattern of alternative haplotypes found in 21p11.2. Distribution of different pairs of haplotypes in each sub-region were shown: (A) B1/B2, (B) D1/D2, and (C) F1/F2.

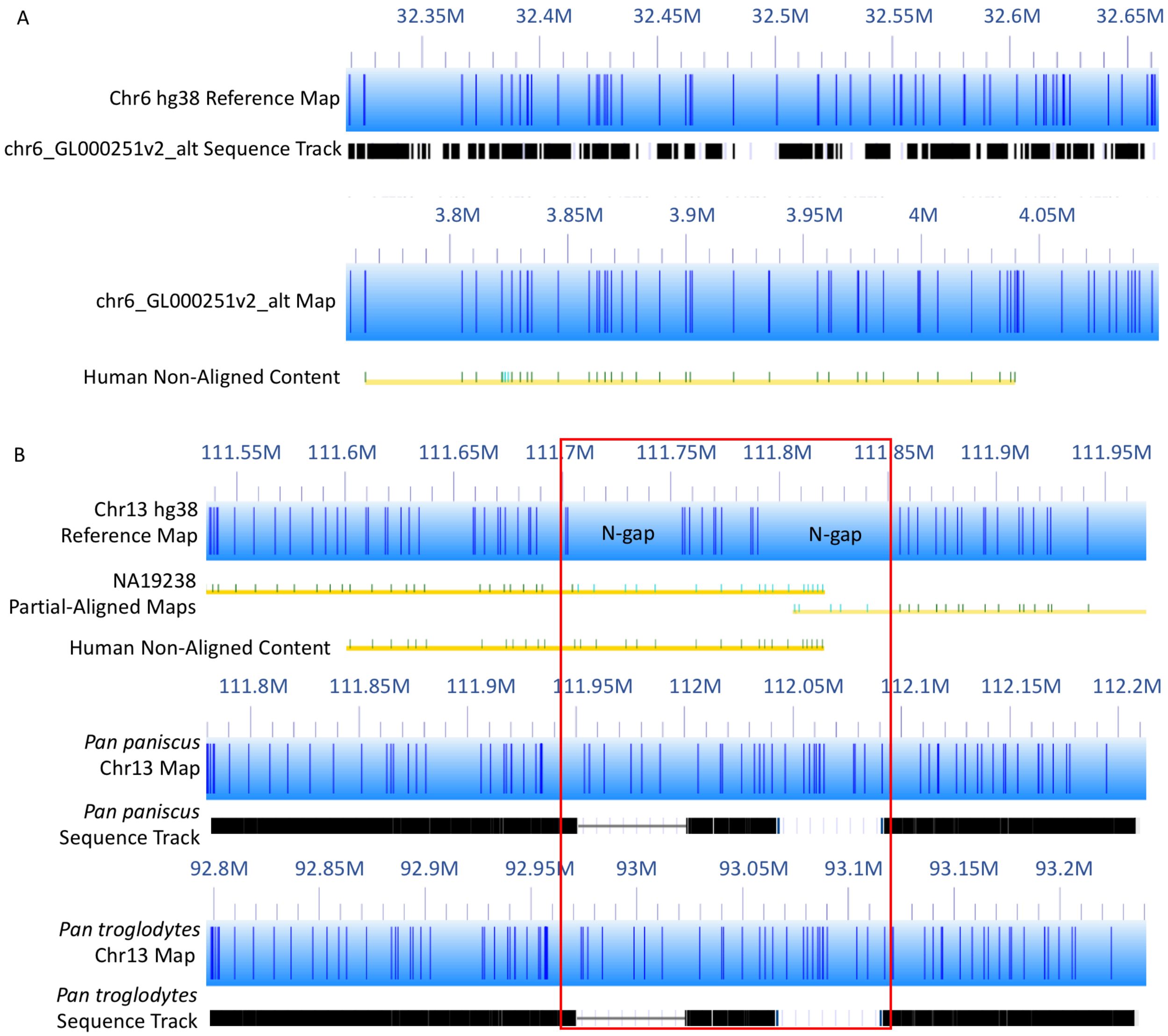

Supplementary Figure 16. Shared non-reference-aligned content with an hg38 alternative sequence and two primate sequences. In (A), non-aligned content (yellow) aligned well with *in silico* nicked chr6_GL000251v2_alt hg38 sequence, which was an alternative sequence of polymorphic MHC class II genes. The chr6_GL000251v2_alt sequence track indicated instances of sequence identity differences (white gaps) compared to the chromosome 6 hg38 reference sequence. Non-aligned content aligned with the alternative sequence, indicating polymorphism with the reference. In (B), *in silico* nicked *Pan paniscus* and *Pan troglodytes* chromosome 13 sequences aligned with non-reference-aligned content. Non-reference content may be contained within non-aligned segments of partially-aligned maps in other genomes, allowing confirmation of maps which should be present in the reference. Hg38 chromosome 13 sequence contained two 50-kb N-gaps, while primate chromosome 13 maps (blue) contained labels in the gaps (red box). The NA19238 partial map, coupled with primate maps, which are highly identical to the hg38 non-gap sequence, indicated our non-reference content as localizing to hg38 chromosome 13 reference gaps.

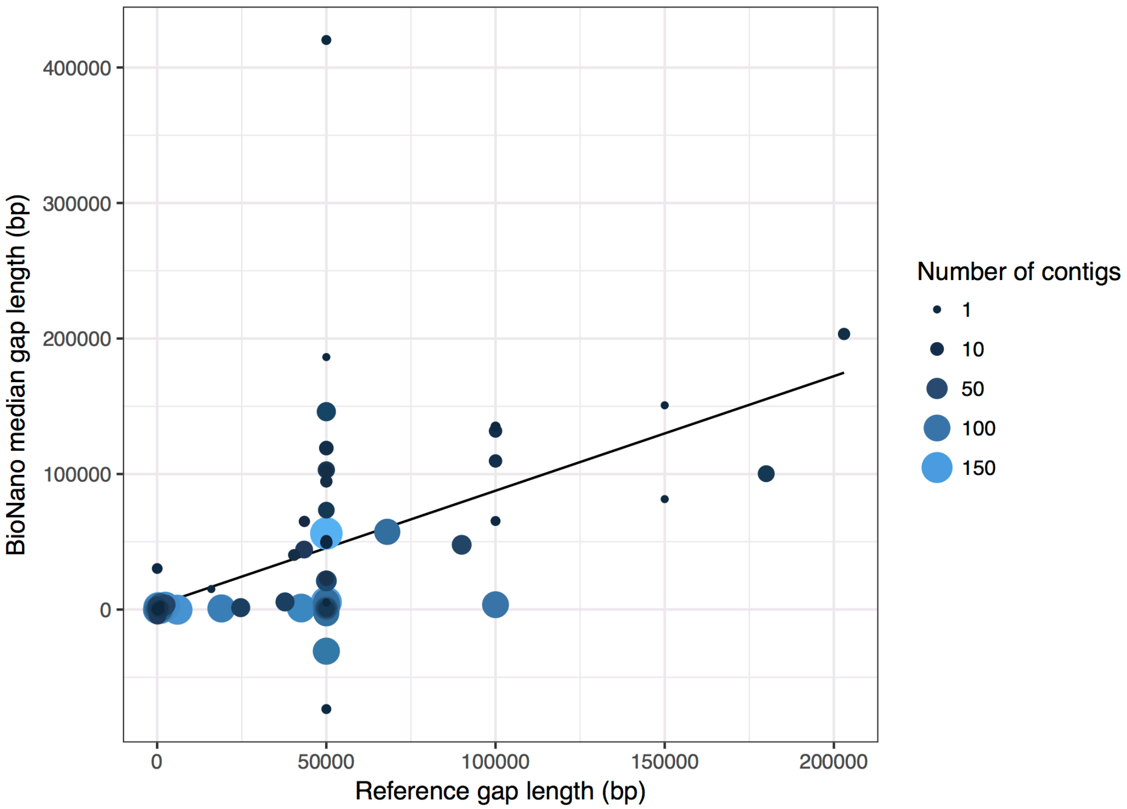

Supplementary Figure 17. Comparison between lengths of reference gaps closed in genome map assemblies (y-axis) and their estimated lengths in the reference genome (x-axis). Each point represents a gap, with size and color representing the number of contigs in which that gap was closed. The diagonal line depicts a linear regression (R^2=0.3).

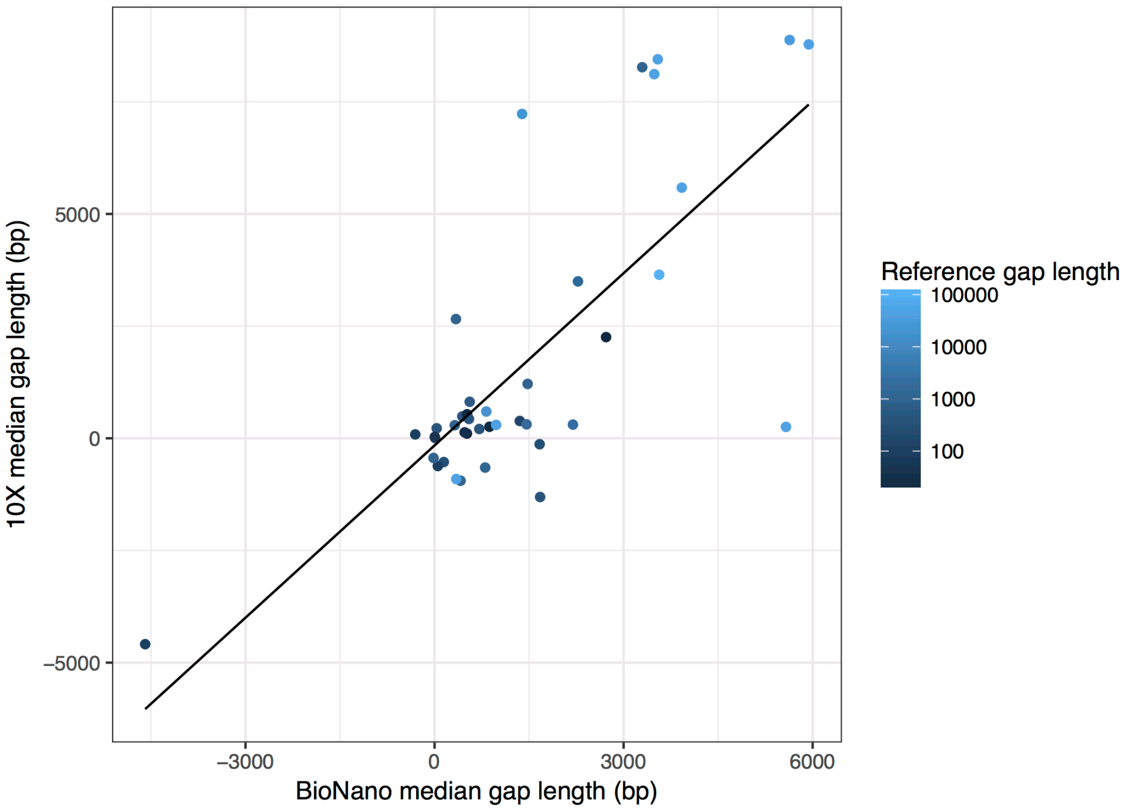

Supplementary Figure 18. Comparison between median lengths of reference gaps closed in 10X assemblies (y-axis) and genome map assemblies (x-axis). Each point represents a gap, with the color representing the estimated length of the gap in the reference genome. The diagonal line depicts a linear regression (R^2=0.56).

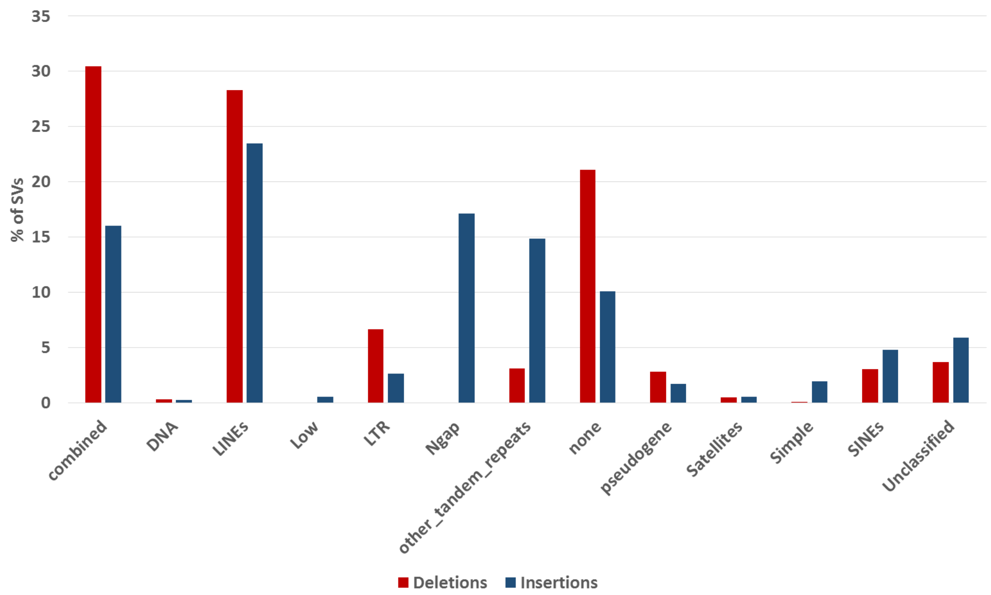

Supplementary Figure 19. Main transposable elements and other sequence repeats found in SVs. SVs were partitioned into sub groups based on their sequence content, when at least 50% of the sequence is of the same repeat class ('none' – SVs with repeat content < 50%, 'combined' – repeat content > 50% but no single class contribute 50% or more to the sequence content)

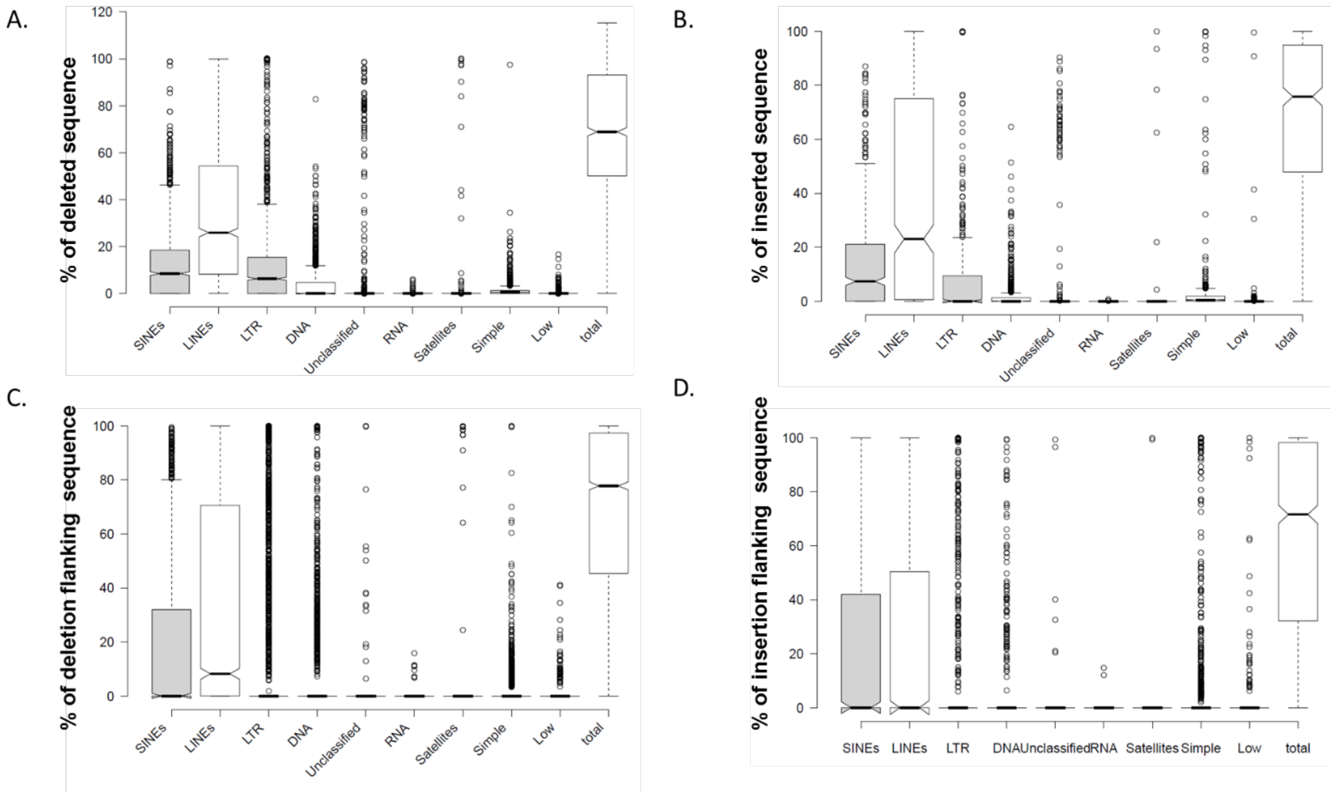

Supplementary Figure 20. Repetitive sequence distribution. As found by RepeatMasker in (A) deletions; (B) insertions; (C) deletion flanking regions; and (D) insertion flanking regions.

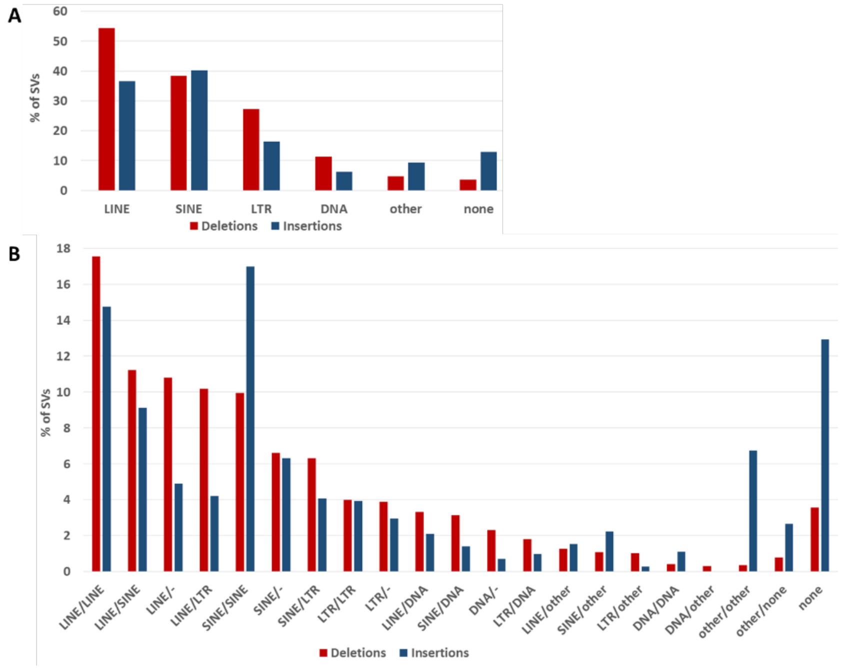

Supplementary Figure 21. Repetitive DNA found in SV flanking regions. A. Percentage of SVs with LINE, SINE, LTR, DNA transposons and other repeats near the SV start and/or end, and B. Percentage of SVs with specific TE combinations near start and end positions.

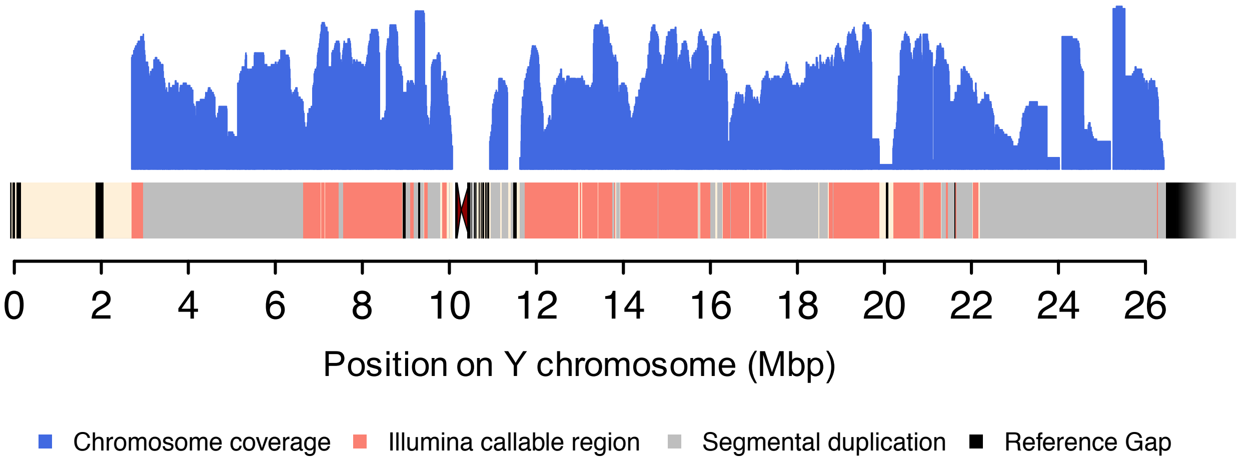

Supplementary Figure 22. Y chromosome assembly overview. The blue histogram above the Y chromosome illustrates the number of male samples whose assemblies aligned to the Y chromosome reference. The chromosome color scheme shows the different properties of the Y chromosome. Salmon, regions of the chromosome where Illumina can access and make variant calls unambiguously; grey, segmental duplications with at least 95% similarity between blocks; black, regions with long sequence- or nick-based gaps in the reference. The variably sized block of heterochromatin on the q arm is not shown in the diagram.

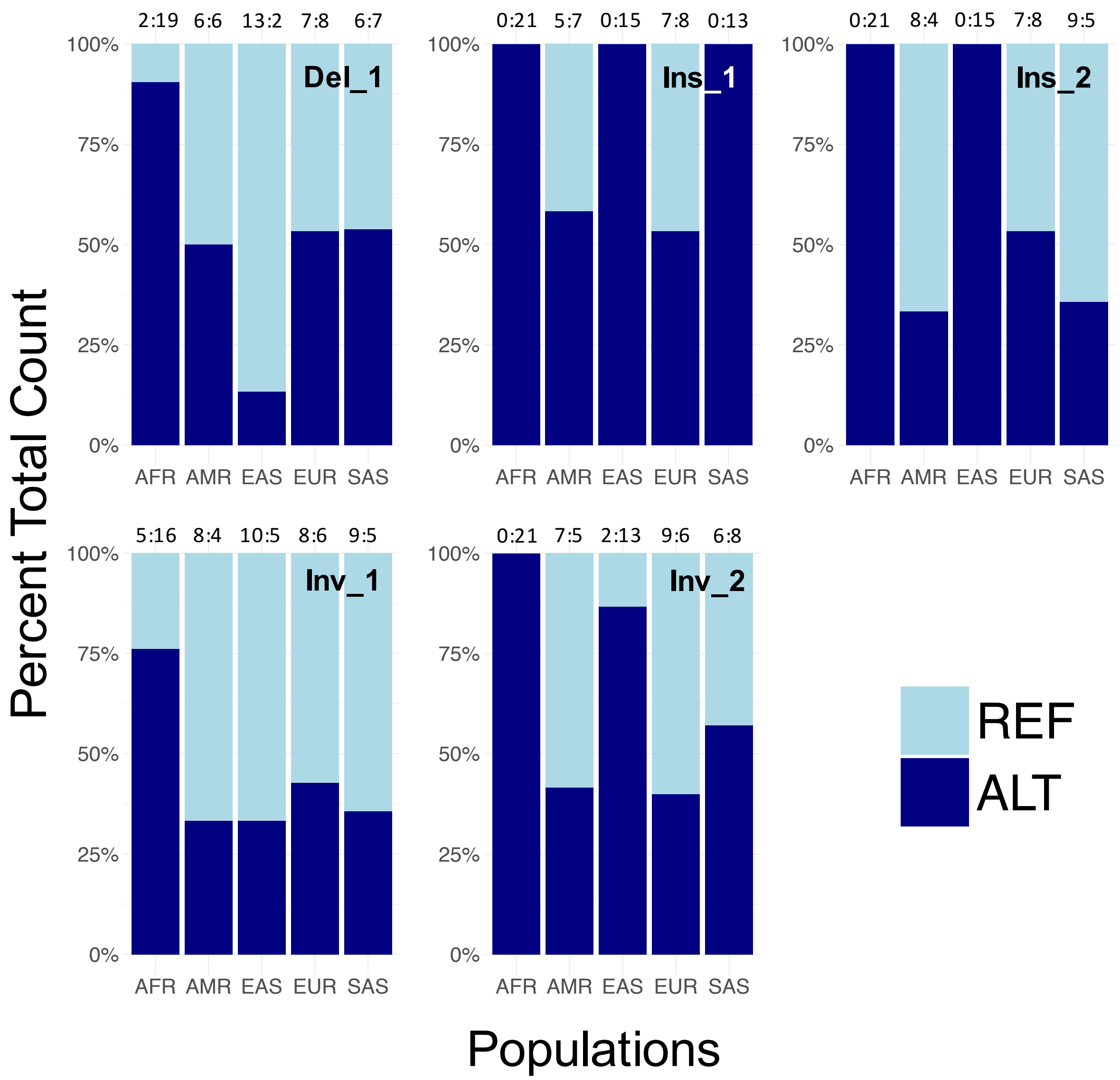

Supplementary Figure 23. Population frequencies of structural variations in the Y chromosome. Stacked bar plot illustrating the individual population proportions of the reference and the non-reference alleles. The numbers on top of each bar represents the actual number of samples supporting either the reference allele (left) or the non-reference allele (right). Super-population abbreviations: AFR - Africans; AMR - Americans; EAS - East Asians; EUR - Europeans; SAS - South Asians. REF – reference allele; ALT – non-reference allele.

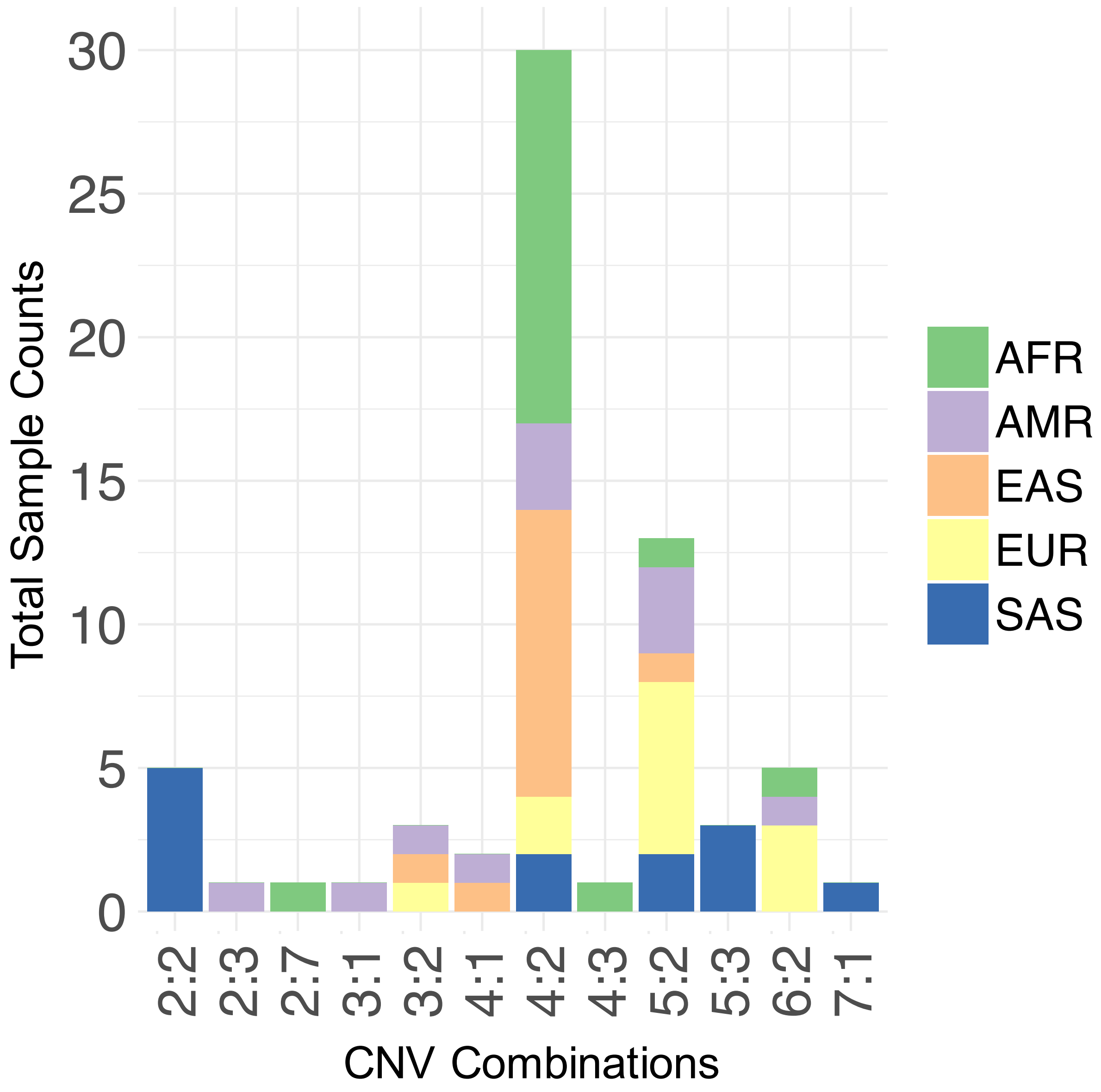

Supplementary Figure 24. Combinations of copy number variations in the Y chromosome. Stacked bar plot showing the different combinations of CNVs found in this study cohort. The x-axis labels show the copy number of CNV_1 (bottom) and CNV_2 (top).

A. Optical maps

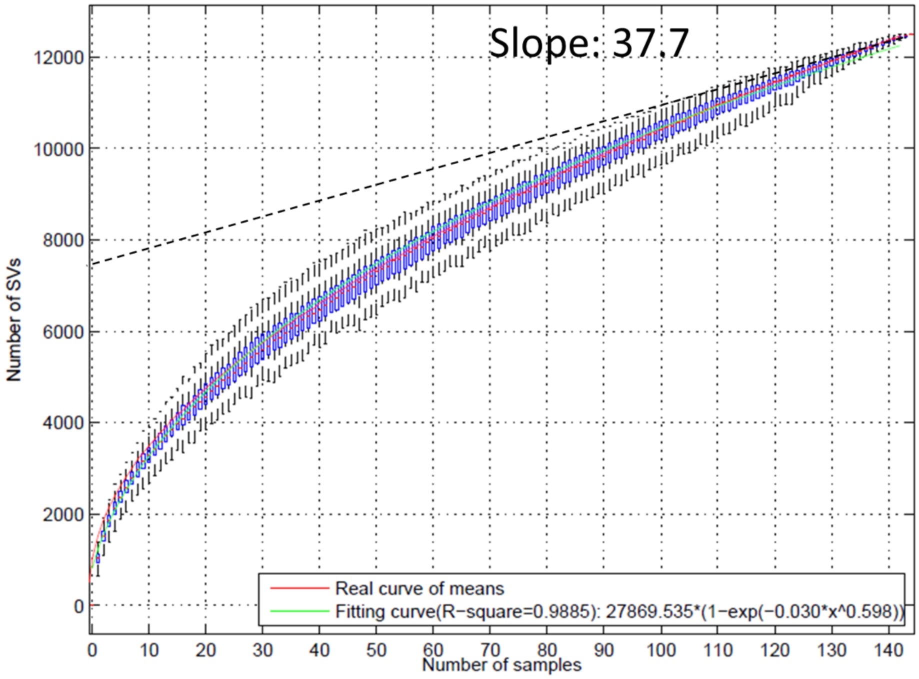

B. 1000 Genomes

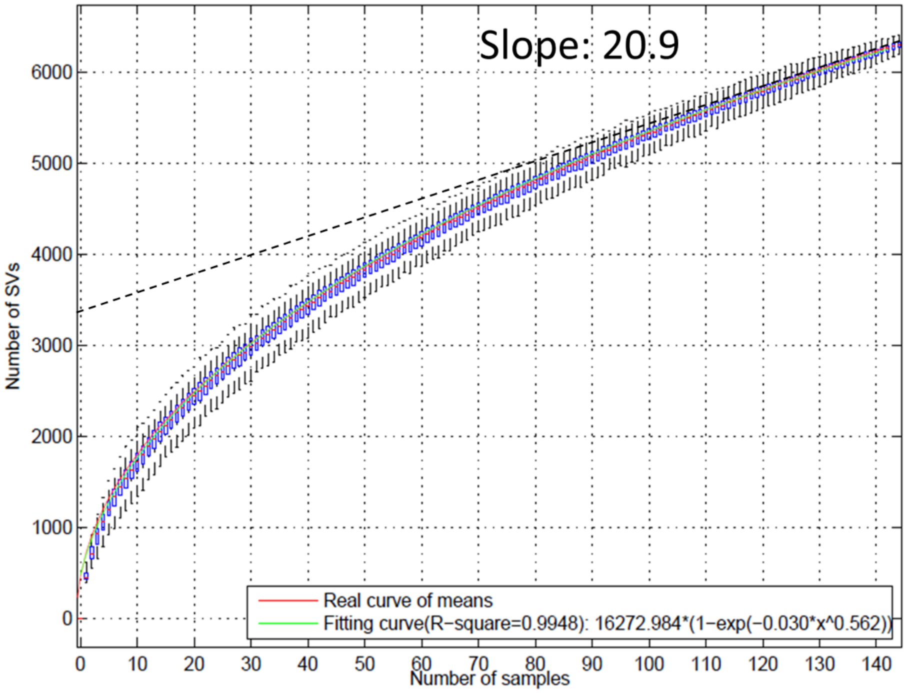

Supplementary Figure 25. Saturation analysis of SVs. The y-axis shows the number of SVs identified if only a given number of samples (specified by the x-axis) are included (A) in this study and (B) in Sudmant et al. (2015)[2], based on the 144 samples commonly studied.

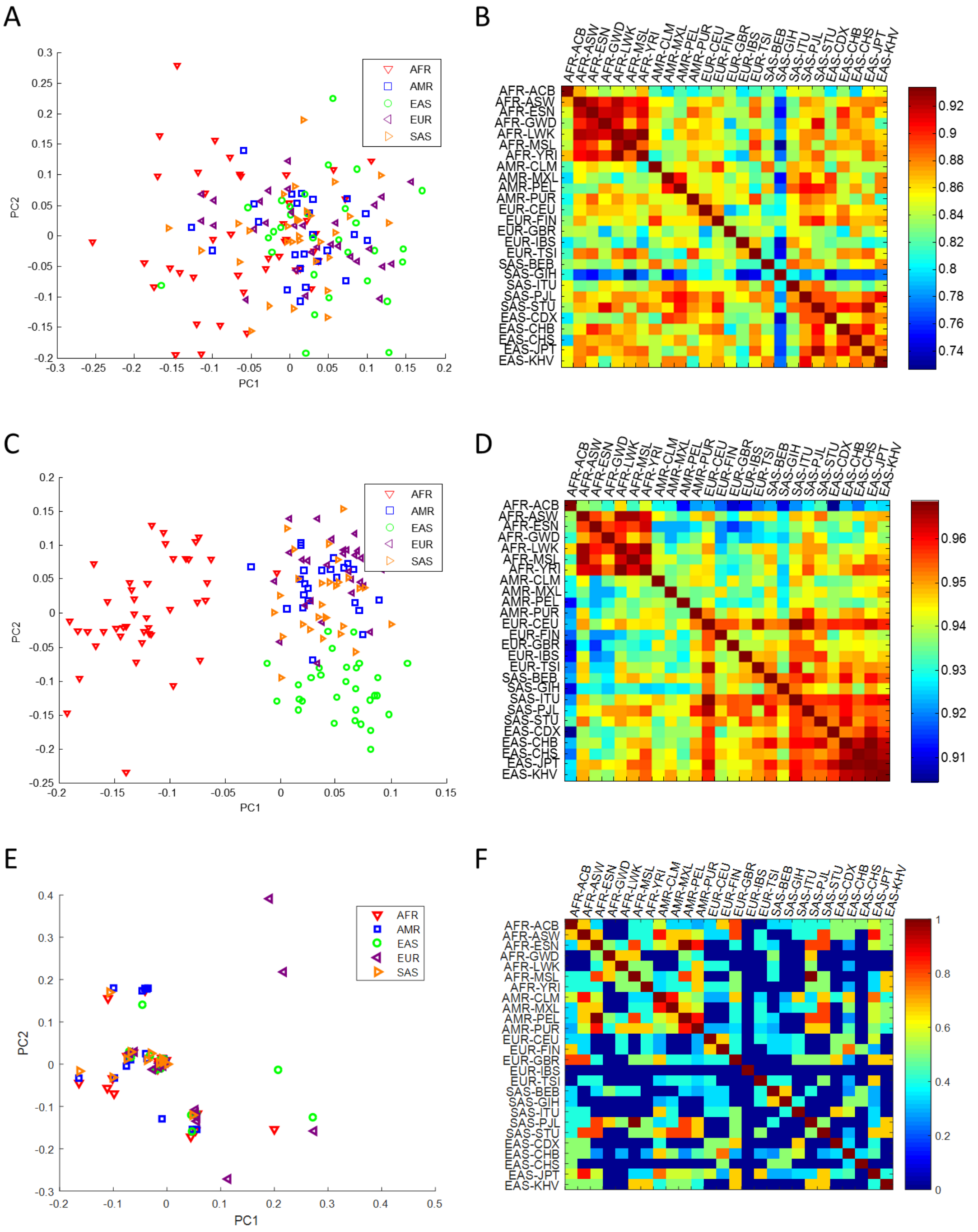

Supplementary Figure 26. Population structure of complex SVs. (A,C,E) The first two principal components of the SV occurrence matrix based on only inversions (A), only loci with multiple indels (C) and only other complex SVs (E). (B,D,F) Heatmaps showing the similarity between different samples based on only inversions (B), only loci with multiple indels (D) and only other complex SVs (F). Super-population abbreviations: AFR - Africans; AMR - Americans; EAS - East Asians; EUR - Europeans; SAS - South Asians.

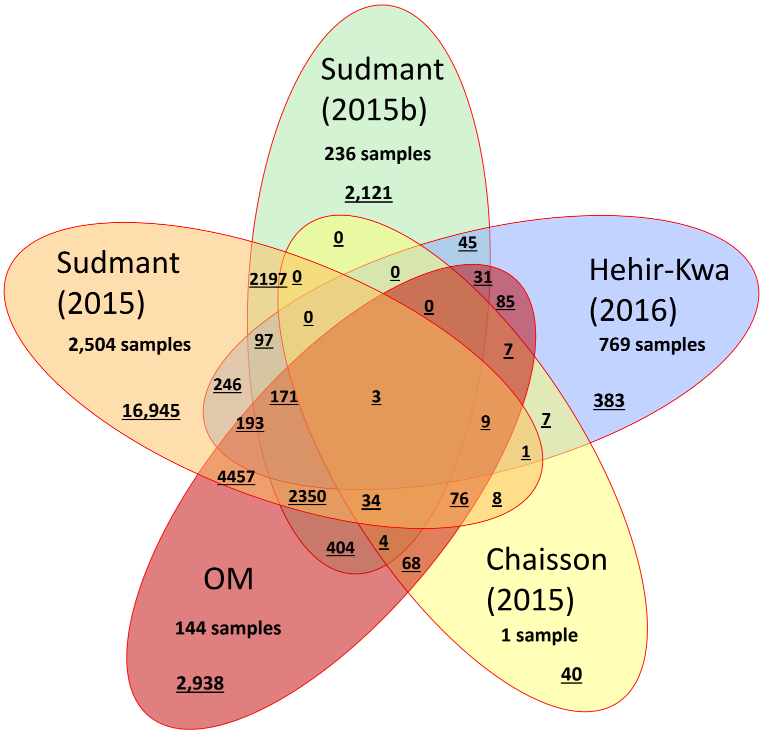

A

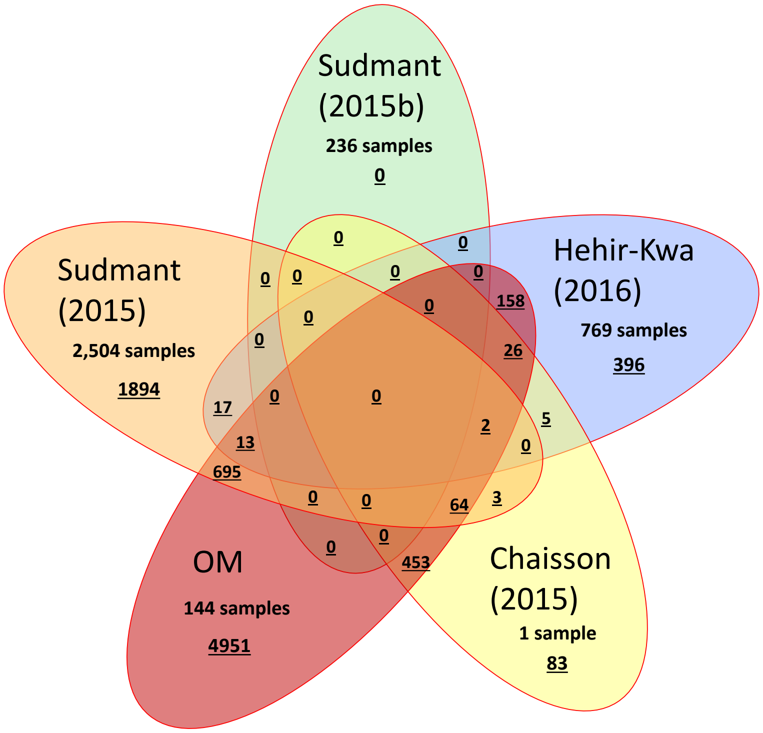

B

Supplementary Figure 27. Complete multi-study Venn diagrams. (A) deletions and (B) insertions. Since one SV in a call set can be verified by multiple SVs in another call set (vise versa), we take the average values for the intersection areas with multiple numbers.

Supplementary Tables

Supplementary Table 1. Summary of hybrid assembly data of individual samples.

Sample	Popu-lation	Super popu-lation	Supernova scaffold N50 (Mb)	Hybrid assembly scaffold N50 (Mb)	Hybrid assembly scaffolds	Hybrid assembly size (Gb)	Total assembly size (Gb)*
HG00250	GBR	EUR	16.8	30.6	232	2.81	2.95
HG00353	FIN	EUR	16.8	27.8	248	2.81	2.94
HG00851	CDX	EAS	18.6	35.4	188	2.81	2.91
HG01971	PEL	AMR	17.9	32.5	208	2.82	2.92
HG02623	GWD	AFR	15.3	27.8	224	2.84	2.96
HG03115	ESN	AFR	17.6	31.9	207	2.81	2.93
HG03838	STU	SAS	23.6	39.2	190	2.80	2.91
NA18552	CHB	EAS	15.9	34.3	216	2.81	2.92
NA19068	JPT	EAS	15.3	29.5	265	2.81	2.92
NA19440	LWK	AFR	19.4	28.7	211	2.80	2.90
NA19789	MXL	AMR	18.3	29.9	226	2.82	2.94
NA20587	TSI	EUR	16.3	27.4	224	2.80	2.91
NA21125	GIH	SAS	16.3	25.4	264	2.80	2.91

* Including Supernova scaffolds that did not contribute to the hybrid assembly

Supplementary Table 2. Validation of SVs identified in the low-complexity regions of the human genome.

Samples	Insertions identified by OMSV	Insertions verifiable by 10x	Insertions supported by 10x	Insertions supported by either 10x or overlapping with 1KG SVs (as a percentage of those verifiable by 10x)		Inserted sequences originate from alternative sequences in hg38	Inserted sequences originate from other locations of the main hg38 reference
HG00250	687	579	395	427	(73.7%)	24	24
HG00353	634	538	346	380	(70.6%)	22	13
HG00851	685	592	405	446	(75.3%)	33	25
HG01971	667	571	404	435	(76.2%)	32	17
HG02623	724	616	440	488	(79.2%)	32	20
HG03115	756	649	417	478	(73.7%)	36	20
HG03838	647	560	408	434	(77.5%)	29	25
NA18552	685	576	403	451	(78.3%)	37	24
NA19068	687	589	410	457	(77.6%)	33	20
NA19440	752	654	448	500	(76.5%)	25	26
NA19789	664	583	417	461	(79.1%)	29	24
NA20587	648	562	390	427	(76.0%)	27	22
NA21125	673	570	385	426	(74.7%)	28	21
Average	685	588	405	447	(76.1%)	30	22
Samples	Deletions identified by OMSV	Deletions verifiable by 10x	Deletions supported by 10x	Deletions supported by either 10x or overlapping with 1KG SVs (as a percentage of those verifiable by 10x)		-	-
HG00250	444	395	335	362	(91.6%)	-	-
HG00353	389	343	302	326	(95.0%)	-	-
HG00851	436	382	341	356	(93.2%)	-	-
HG01971	441	384	329	363	(94.5%)	-	-
HG02623	542	477	451	466	(97.7%)	-	-
HG03115	542	453	432	441	(97.4%)	-	-
HG03838	475	424	368	399	(94.1%)	-	-
NA18552	437	385	358	374	(97.1%)	-	-
NA19068	415	375	342	356	(94.9%)	-	-
NA19440	497	449	417	433	(96.4%)	-	-
NA19789	424	370	339	356	(96.2%)	-	-
NA20587	394	355	333	341	(96.1%)	-	-
NA21125	411	365	320	345	(94.5%)	-	-
Average	450	397	359	378	(95.4%)	-	-

Supplementary Table 3. Concordance rate of SVs identified from 4 family trios

Trio	Samples	Number of SVs identified	Concordant with Mendelian inheritance	
1	HG00512, HG00513, HG00514	1,810	1,760	(97.2%)
2	HG00731, HG00732, HG00733	1,560	1,328	(85.1%)
3	NA12891, NA12892, NA12878	1,566	1,516	(96.8%)
4	NA19238, NA19239, NA19240	2,819	2,792	(99.0%)
Total		7,755	7,396	(95.4%)

Supplementary Table 4. GWAS entries in analysed CNV regions.

CNV loci	CNV region coordinates	GWAS Entries (NHGRI-EBI Catalog)			
		**Position**	**Reported Gene(s)**	**Context**	**Mapped Trait**
1q23.1	1:161545097-161626543	1:161549650	FCGR2A, RP11-25K21.6, HSPA6, RPS23P10, FCGR3A	intron_variant	low affinity immunoglobulin gamma Fc region receptor II-a/b measurement
		1:161571067	FCGR2A, FCGR2B	intron_variant	lipid measurement
8q21	8:7100000-8200000	8:7141613	DEFA5, LOC349196	intron_variant	p-tau:beta-amyloid 1-42 ratio measuremen
		8:7331836	LOC401447	intron_variant	optic disc size measurement
16q22.2	16:72054626-72076832	16:72074194	HP, HPR, DHX38, TXNL4B, PMFBP1	intron_variant	haptoglobin measurement
		16:72074194	HP, HPR, DHX38, TXNL4B, PMFBP1	intron_variant	hemoglobin measurement
		16:72074194	HP, HPR, DHX38, TXNL4B, PMFBP1	intron_variant	heparin cofactor 2 measurement
		16:72074194	HPR, HP, DHX38	intron_variant	low density lipoprotein cholesterol measurement
		16:72074194	HPR, HP, DHX38	intron_variant	total cholesterol measurement
17p11.2	17:18900000-19200000	17:18941423		upstream_gene_variant	ankle injury
		17:19008954		intron_variant	schizophrenia, response to paliperidone, schizophrenia symptom severity measurement
		17:19020368	SLC5A10	synonymous_variant	1,5 anhydroglucitol measurement

Supplementary Table 5. List of non-aligned human genome content mapped to non-human primates.

Human Sample #	Contig_Start	Contig_End	Query Length	Primate_Ref Start	Primate_Ref End	Primate_Ref Length	Primate_Genbank	Primate_Name_Chromosome
GM19921	646763.7	20	646743.7	30192695	30828559	635864	GCA_000146795.3_Nleu_3.0	CM001649.1 Nomascus leucogenys chromosome 3
GM18623	523823.4	20	523803.4	40650185	41136888	486703	GCA_000151905.3_gorGor4	FR853095.2 Gorilla gorilla gorilla genomic chromosome, chr2B
GM19920	20	480153.8	480133.8	89574059	90028970	454911	GCA_000151905.3_gorGor4	FR853095.2 Gorilla gorilla gorilla genomic chromosome, chr2B
HG03740	20	321783.9	321763.9	110386720	110679639	292919	GCA_000151905.3_gorGor4	FR853095.2 Gorilla gorilla gorilla genomic chromosome, chr2B
GM21137	20	479961.2	479941.2	41387065	41849380	462315	GCA_000151905.3_gorGor4	FR853098.3 Gorilla gorilla gorilla genomic chromosome, chr5
GM11994	502673.1	20	502653.1	130091699	130569437	477738	GCA_000151905.3_gorGor4	FR853100.2 Gorilla gorilla gorilla genomic chromosome, chr7
GM19921	20	646763.7	646743.7	118117643	118745037	627394	GCA_000151905.3_gorGor4	FR853081.2 Gorilla gorilla gorilla genomic chromosome, chr10
HG01816	20	220096.2	220076.2	88226294	88441899	215605	GCA_000151905.3_gorGor4	FR853085.2 Gorilla gorilla gorilla genomic chromosome, chr14
NA19678	570189.5	20	570169.5	14201998	14723282	521284	GCA_000151905.3_gorGor4	FR853088.3 Gorilla gorilla gorilla genomic chromosome, chr17

NA19720	522080.6	32000.2	490080.4	18843628	19268928	425300	GCA_000151905.3_gorGor4	FR853090.2 Gorilla gorilla gorilla genomic chromosome, chr19
GM18623	523823.4	20	523803.4	152960155	153453234	493079	GCA_000258655.2_panpan1.1	CM003385.1 Pan paniscus isolate Ulindi chromosome 2B
GM19920	6670.2	480153.8	473483.6	202007287	202449853	442566	GCA_000258655.2_panpan1.1	CM003385.1 Pan paniscus isolate Ulindi chromosome 2B
HG03740	20	321783.9	321763.9	223044287	223336163	291876	GCA_000258655.2_panpan1.1	CM003385.1 Pan paniscus isolate Ulindi chromosome 2B
HG02107	435020.5	5119.3	429901.2	18784536	19188572	404036	GCA_000258655.2_panpan1.1	CM003386.1 Pan paniscus isolate Ulindi chromosome 3
GM19914	20	410947.2	410927.2	113603296	113974767	371471	GCA_000258655.2_panpan1.1	CM003387.1 Pan paniscus isolate Ulindi chromosome 4
GM11994	502673.1	20	502653.1	135262386	135732906	470520	GCA_000258655.2_panpan1.1	CM003390.1 Pan paniscus isolate Ulindi chromosome 7
GM19921	20	646763.7	646743.7	103512887	104140923	628036	GCA_000258655.2_panpan1.1	CM003393.1 Pan paniscus isolate Ulindi chromosome 10
HG01816	20	220096.2	220076.2	104534248	104741329	207081	GCA_000258655.2_panpan1.1	CM003397.1 Pan paniscus isolate Ulindi chromosome 14
GM21137	20	479961.2	479941.2	15509456	15963601	454145	GCA_000258655.2_panpan1.1	CM003400.1 Pan paniscus isolate Ulindi chromosome 17
NA19678	20	570189.5	570169.5	41822931	42378011	555080	GCA_000258655.2_panpan1.1	CM003400.1 Pan paniscus isolate Ulindi chromosome 17

NA19720	522080.6	20	522060.6	19425558	19874011	448453	GCA_000258655.2_panpan1.1	CM003402.1 Pan paniscus isolate Ulindi chromosome 19
GM21095	393758.7	20	393738.7	22587595	22976361	388766	GCA_000258655.2_panpan1.1	CM003405.1 Pan paniscus isolate Ulindi chromosome 22
GM21095	20	262426.2	262406.2	14882741	15144868	262127	GCA_000258655.2_panpan1.1	CM003406.1 Pan paniscus isolate Ulindi chromosome X
GM18623	523823.4	20	523803.4	35162416	35655671	493255	GCA_002880755.3_Clint_PTRv2	CM009240.2 Pan troglodytes isolate Yerkes chimp pedigree #C0471 (Clint) chromosome 2B
GM19920	6670.2	480153.8	473483.6	83436659	83882632	445973	GCA_002880755.3_Clint_PTRv2	CM009240.2 Pan troglodytes isolate Yerkes chimp pedigree #C0471 (Clint) chromosome 2B
HG03740	20	321783.9	321763.9	103831750	104124010	292260	GCA_002880755.3_Clint_PTRv2	CM009240.2 Pan troglodytes isolate Yerkes chimp pedigree #C0471 (Clint) chromosome 2B
HG02107	435020.5	5119.3	429901.2	18718278	19126635	408357	GCA_002880755.3_Clint_PTRv2	CM009241.2 Pan troglodytes isolate Yerkes chimp pedigree #C0471 (Clint) chromosome 3
GM19914	20	410947.2	410927.2	108125610	108498624	373014	GCA_002880755.3_Clint_PTRv2	CM009242.2 Pan troglodytes isolate Yerkes chimp pedigree #C0471 (Clint) chromosome 4
HG01784	284730.9	20	284710.9	58731232	59013581	282349	GCA_002880755.3_Clint_PTRv2	CM009243.2 Pan troglodytes isolate Yerkes chimp pedigree #C0471 (Clint) chromosome 5

GM11994	502673.1	20	502653.1	127680551	128152609	472058	GCA_002880755.3_Clint_PTRv2	CM009245.2 Pan troglodytes isolate Yerkes chimp pedigree #C0471 (Clint) chromosome 7
GM19921	20	646763.7	646743.7	99296402	99932356	635954	GCA_002880755.3_Clint_PTRv2	CM009248.2 Pan troglodytes isolate Yerkes chimp pedigree #C0471 (Clint) chromosome 10
NA19678	20	440718.6	440698.6	28677291	29081632	404341	GCA_002880755.3_Clint_PTRv2	CM009251.2 Pan troglodytes isolate Yerkes chimp pedigree #C0471 (Clint) chromosome 13
GM21137	20	479961.2	479941.2	14761411	15216297	454886	GCA_002880755.3_Clint_PTRv2	CM009255.2 Pan troglodytes isolate Yerkes chimp pedigree #C0471 (Clint) chromosome 17
NA19678	20	570189.5	570169.5	36334755	36847558	512803	GCA_002880755.3_Clint_PTRv2	CM009255.2 Pan troglodytes isolate Yerkes chimp pedigree #C0471 (Clint) chromosome 17
NA19720	522080.6	20	522060.6	19513214	19968798	455584	GCA_002880755.3_Clint_PTRv2	CM009257.2 Pan troglodytes isolate Yerkes chimp pedigree #C0471 (Clint) chromosome 19
GM21095	393758.7	20	393738.7	6522709	6910431	387722	GCA_002880755.3_Clint_PTRv2	CM009260.2 Pan troglodytes isolate Yerkes chimp pedigree #C0471 (Clint) chromosome 22
GM21095	20	262426.2	262406.2	14935461	15198966	263505	GCA_002880755.3_Clint_PTRv2	CM009261.2 Pan troglodytes isolate Yerkes chimp pedigree #C0471 (Clint) chromosome X

GM18623	523823.4	20	523803.4	34394515	34893217	498702	GCA_002880 775.3_Susie_ PABv2	CM009264.2 Pongo abelii isolate Susie chromosome 2B
GM11994	494001.2	20	493981.2	116731531	117195699	464168	GCA_002880 775.3_Susie_ PABv2	CM009269.2 Pongo abelii isolate Susie chromosome 7
GM19921	20	646763.7	646743.7	101817564	102444912	627348	GCA_002880 775.3_Susie_ PABv2	CM009272.2 Pongo abelii isolate Susie chromosome 10
NA19720	522080.6	20	522060.6	19182088	19639235	457147	GCA_002880 775.3_Susie_ PABv2	CM009281.2 Pongo abelii isolate Susie chromosome 19

Supplementary Table 6. Reference gaps closed with genome

Gap coordinates	Reference gap length (bp)	Number of genome map contigs closing gap	Mean closed gap length (bp)	Median closed gap length (bp)
chr1:124977944-124978326	382	1	34	34
chr1:125013060-125013223	163	1	1353	1353
chr1:125103213-125103233	20	1	514	514
chr1:12954384-13004384	50000	15	22901.5	22930
chr1:16799163-16849163	50000	36	154611.9	146016
chr1:223558935-223608935	50000	68	1014.7	977.5
chr1:228558364-228608364	50000	16	104594.4	102737.5
chr1:2702781-2746290	43509	4	70752.8	65034
chr1:29552233-29553835	1602	139	1551.5	1460
chr10:124121200-124121502	302	8	134.2	146.5
chr10:131597030-131597130	100	26	-4583.8	-4592
chr10:133690466-133740466	50000	91	5025.8	-2860
chr10:38529907-38573338	43431	28	44061.4	44259
chr10:39254773-39254793	20	3	846.7	871
chr10:39409792-39410237	445	3	598.3	440
chr10:39479351-39479371	20	2	517	517
chr10:39497198-39497296	98	2	-306.5	-306.5
chr10:47780368-47870368	90000	40	47766.9	47771
chr11:70955696-71055696	100000	101	3572.5	3565
chr11:87978202-88002896	24694	35	1352	1389

chr11:96566178-96566364	186	117	117.2	124
chr12:132223362-132224362	1000	60	3436.2	3819.5
chr12:37379851-37380460	609	43	498.5	558
chr12:37460032-37460128	96	61	164	-9
chr12:7083650-7084650	1000	135	510.4	413
chr13:113673020-113723020	50000	22	597.5	640.5
chr14:18712644-18862644	150000	1	81474	81474
chr14:19511713-19611713	100000	9	134466.9	131740
chr15:20689304-20729746	40442	5	40125	40259
chr15:23226874-23276874	50000	25	90869.9	103014
chr16:33392411-33442411	50000	6	49441	49232
chr17:22089188-22089410	222	85	24.3	42
chr17:22763679-22813679	50000	1	5172	5172
chr17:26698590-26698998	408	5	578.2	710
chr17:26720420-26721376	956	15	1479.2	1477
chr17:26735204-26735774	570	23	14.1	9
chr17:81742542-81792542	50000	99	347.3	344
chr18:46969912-47019912	50000	170	55562.6	56128.5
chr18:54536574-54537528	954	119	733.4	803
chr2:16145119-16146119	1000	1	3295	3295
chr2:238903659-238904047	388	140	1649.2	1675.5
chr2:89685992-89753992	68000	94	57349.7	57341

chr2:94293015-94496015	203000	6	203125.8	203346
chr2:97439618-97489618	50000	47	995.6	989
chr20:29315342-29315821	479	36	513.8	546.5
chr20:29362154-29362183	29	41	484.2	477
chr20:29412507-29413577	1070	29	323.4	321
chr20:29447838-29447883	45	31	-5.6	4
chr20:29452158-29452178	20	35	2722.3	2722
chr20:29556103-29556141	38	85	137.8	138
chr20:29592644-29592737	93	125	-74.6	-79
chr20:29651590-29651610	20	111	160.7	-102
chr20:29697363-29697630	267	124	786.2	1667.5
chr21:41584292-41584392	100	95	53.6	52
chr21:43212462-43262462	50000	151	6078.9	5577
chr21:8706715-8756715	50000	2	420287	420287
chr21:8886604-8986604	100000	2	135066	135066
chr21:9196087-9246087	50000	4	65046.5	50851.5
chr21:9377143-9527143	150000	1	150725	150725
chr22:18239129-18339129	100000	9	109699	109646
chr22:18433513-18483513	50000	6	94584.2	94539
chr22:18659564-18709564	50000	1	186312	186312
chr22:49973865-49975365	1500	57	2275.5	2276
chrX:114281198-114331198	50000	92	5905.6	5938.5

chrX:115738949-115838949	100000	2	65331.5	65331.5
chrX:116557779-116595566	37787	35	5547.2	5635
chrX:120879381-120929381	50000	104	-30364.4	-30736
chrX:144425606-144475606	50000	114	3506	3542.5
chrX:226276-226351	75	3	98.7	186
chrX:49348394-49528394	180000	23	100933.5	100253
chrX:50228964-50278964	50000	102	3921.9	3923.5
chrY:20207793-20257793	50000	2	-73445	-73445
chrY:21789281-21805281	16000	1	15126	15126
chrY:9403713-9453713	50000	22	66824.9	73354
chr3:91345078-91345173	95	31	187.5	204
chr3:91364131-91364151	20	34	228.4	233.5
chr3:91438798-91438818	20	26	239.7	230.5
chr4:190123121-190173121	50000	6	49191.7	49953.5
chr4:32833016-32839016	6000	138	-114.3	-102
chr4:58878793-58921381	42588	123	1068	1104
chr4:8797477-8816477	19000	115	817.1	821
chr4:9272916-9322916	50000	47	20695.8	21172
chr5:139452659-139453659	1000	73	413.3	339
chr5:155760324-155761324	1000	77	-19.6	-16
chr5:17530548-17580548	50000	13	112779.4	119144
chr6:95020790-95070790	50000	91	3914.1	3486
chr7:143650804-143700804	50000	5	102668	102683

chr7:237846-240242	2396	133	2248.7	2196
chr8:85664222-85714222	50000	1	51716	51716
chr9:134183092-134185536	2444	43	3699.5	3496
chr9:43240559-43240579	20	3	30231.3	30275
chr9:65595191-65645191	50000	2	48921.5	48921.5

Supplementary Table 7. List of structural variations found in the human Y chromosome.

SV	type	chr	start	end	size_change (bp)	overlapping genes	sequence class
Del_1	deletion	Y	6,679,977	6,727,439	-15788	NA	X-transposed Region
Ins_1	insertion	Y	7,709,611	7,723,973	3,710	NA	X-degenerate Region
Del_2	deletion	Y	9,044,419	9,149,320	-50,007	NA	Assembly gap
Ins_2	insertion	Y	9,907,429	9,913,864	19,842	NA	Ampliconic
Inv_1	inversion	Y	16,261,115	16,324,202	NA	NA	Ampliconic
Inv_2	inversion	Y	21,020,262	21,053,760	NA	NA	Ampliconic
CNV_1	copy number variation	Y	21,482,338	21,565,233	varies	RBMY1A1; RBMY1B	Ampliconic
Del_3	deletion	Y	21,738,492	21,808,848	-40,109	NA	Ampliconic
CNV_2	copy number variation	Y	21,864,175	21,947,925	varies	RBMY1D; RBMY1E	Ampliconic

Supplementary Table 8. Y chromosome CNV statistics of individual male samples.

sample	super_pop	ROI_6_CNV	ROI_8_CNV
HG00101	EUR	1	no_reliable_molecule
HG00251	EUR	5	2
HG00260	EUR	5	2
HG00271	EUR	6	2
HG00329	EUR	6	2
HG00351	EUR	6	2
HG00472	EAS	4	2
HG00512	EAS	4	2
HG00622	EAS	4	2
HG00731	AMR	5	2
HG00844	EAS	4	2
HG01133	AMR	4	2
HG01139	AMR	4	2
HG01167	AMR	2	3
HG01176	AMR	5	2
HG01464	AMR	6	2
HG01756	EUR	4	2
HG01761	EUR	5	2
HG01783	EUR	no_reliable_molecule	no_reliable_molecule
HG01816	EAS	2	no_reliable_molecule
HG01873	EAS	4	2
HG01970	AMR	3	2
HG01974	AMR	4	1
HG01991	AMR	4	2
HG02026	EAS	no_reliable_molecule	2
HG02107	AFR	4	2
HG02250	EAS	4	2
HG02283	AFR	4	2
HG02332	AFR	4	2
HG02490	SAS	2	2
HG02521	EAS	4	2
HG02594	AFR	4	2
HG02603	SAS	5	3
HG02623	AFR	6	2
HG02687	SAS	no_reliable_molecule	3
HG02810	AFR	5	2
HG03006	SAS	4	2
HG03096	AFR	no_reliable_molecule	3

HG03115	AFR	4	2
HG03124	AFR	4	2
HG03133	AFR	4	2
HG03451	AFR	4	2
HG03469	AFR	no_reliable_molecule	2
HG03615	SAS	4	2
HG03682	SAS	5	2
HG03725	SAS	5	2
HG03727	SAS	2	2
HG03740	SAS	5	3
HG03797	SAS	7	1
HG03864	SAS	2	2
HG04006	SAS	2	2
NA06986	EUR	5	2
NA12342	EUR	5	2
NA12891	EUR	no_reliable_molecule	no_reliable_molecule
NA18486	AFR	4	2
NA18557	EAS	5	2
NA18622	EAS	4	2
NA18623	EAS	3	2
NA18986	EAS	4	1
NA18988	EAS	4	2
NA19025	AFR	4	2
NA19068	EAS	4	2
NA19213	AFR	4	2
NA19239	AFR	2	7
NA19443	AFR	4	2
NA19444	AFR	no_reliable_molecule	2
NA19720	AMR	5	no_reliable_molecule
NA19789	AMR	5	2
NA19795	AMR	3	1
NA19920	AFR	4	2
NA19982	AFR	0	no_reliable_molecule
NA19984	AFR	4	3
NA20588	EUR	3	2
NA20814	EUR	5	2
NA20815	EUR	4	2
NA21095	SAS	5	3
NA21126	SAS	2	2

Supplementary Table 9: Comparative analysis of inversions with the Strand-seq callset

Categories of inversions in Strand-seq callset	Found in our list	Close to (<50kb) /including gaps of HG38	Not found in our list	Total
**Number of cases**	72	12	15	99
**Average count of Inversion alleles (=#passing cells * InvFreq * 2)**	12.9	12.6	10	12.4
**Average frequency of inversion alleles**	0.36	0.33	0.25	0.34
**Average number of DGV hits**	6.4	2.5	3.2	5.4
**Proportion of inversions with DGV hits**	81%	25%	33%	67%

Supplementary Table 10: Case studies of strand-seq inversions (lifted over from hg19 to hg38)

#Chr	start	stop	Found in Supplementary Table 2?	Marks of closing gaps (within 50kb distance)	Passing cells	InvFreq	Number of inversion alleles (=#passing cells * InvFreq * 2)	Number of DGV hits
chr1	108311626	108379425	Yes		7	0.57	8	5
chr1	143773200	143822242	Yes		12	0.25	6	3
chr2	89870185	90229790	Yes		21	0.05	2	4
chr2	91837395	92014973	Yes		20	0.25	10	0
chr2	92082813	92131696	Close to/including gaps	Close to the gap chr2:92138145-92188145	21	0.31	13	0
chr2	95464814	95597442	Yes		17	0.24	8	7
chr2	110095657	110305950	Yes		21	0.86	36	0
chr3	195662291	195997499	Yes		24	0.25	12	11
chr4	10001	69199	Close to/including gaps	Close to the gap chr4:0-10000	24	0.4	19	0
chr4	189618757	189762610	Close to/including gaps	Close to the gap chr4:190123121-190173121	24	0.4	19	16
chr4	190096468	190123121	Close to/including gaps	Close to the gap chr4:190123121-190173121	13	0.38	10	0
chr5	21463893	21590485	No		20	0.08	3	2
chr5	69620537	71349741	Yes		20	0.38	15	8
chr5	177724584	177907642	Yes		16	0.25	8	6
chr6	273491	381214	No		17	0.29	10	0
chr6	60408914	60641599	No		17	0.15	5	2
chr7	5981622	6739366	Yes		19	0.16	6	5
chr7	6739366	6825376	Yes		7	0.36	5	3
chr7	54234757	54308696	Yes		12	0.58	14	18
chr7	56789081	57054984	No		18	0.14	5	0
chr7	57636060	57839116	Yes		18	0.33	12	1
chr7	63365457	63699036	No		18	0.28	10	2
chr7	65118381	65547885	Yes		19	0.34	13	3
chr7	65547990	65648089	Yes		9	0.22	4	6
chr7	143724405	143880519	Yes		13	0.42	11	17
chr7	152382337	152416239	No		13	0.31	8	0
chr8	8198267	12123140	Yes		20	0.22	9	18
chr9	66756492	67217003	Close to/including gaps	Close to the gap chr9:66391387-66591387	26	0.75	39	0
chr9	61518808	61700455	Yes		25	0.08	4	2
chr9	61822141	62114051	Yes		8	0.5	8	0
chr9	41754713	41912138	Yes		18	0.56	20	0

chr9	40711178	40910315	Yes		26	0.77	40	5
chr9	40929925	41103545	Yes		23	0.89	41	1
chr9	64315967	64998124	Yes		26	0.63	33	4
chr10	47647974	47739029	Yes		7	0.29	4	7
chr10	79518741	80265251	Yes		21	0.12	5	0
chr11	48319210	48365199	No		19	0.39	15	0
chr11	50134285	50347953	Yes		26	0.33	17	2
chr11	89823584	90071086	Yes		17	0.35	12	9
chr12	10000	45738	Close to/including gaps	Close to the gap chr12:0-10000	6	0.33	4	0
chr12	131284999	131701921	Yes		21	0.1	4	4
chr14	18223524	18352647	Close to/including gaps	Close to the gap chr14:18173523-18223523	21	0.26	11	0
chr14	19249376	19951026	Yes		21	0.33	14	5
chr15	19794747	20181509	Yes		23	0.28	13	3
chr15	21974163	22308242	Yes		23	0.39	18	20
chr15	30147796	30569812	Yes		19	0.29	11	1
chr15	30603602	32167290	Yes		23	0.07	3	2
chr15	32199642	32454146	Yes		14	0.25	7	2
chr15	84234733	84405300	Close to/including gaps	Including the gap chr15:84270066-84320066	13	0.46	12	6
chr15	101948809	101981189	Close to/including gaps	Close to the gap chr15:101981189-101991189	6	0.25	3	0
chr16	14799891	14919808	Yes		13	0.81	21	27
chr16	15031827	15332486	Yes		21	0.79	33	4
chr16	16295521	16489142	No		13	0.19	5	0
chr16	16547971	18125361	Yes		22	0.05	2	3
chr16	18119815	18732060	Yes		22	0.11	5	3
chr16	21506590	21592603	Yes		20	0.45	18	31
chr16	21595911	21738398	Yes		22	0.86	38	31
chr16	21790408	22485813	Yes		22	0.82	36	31
chr16	28413453	28777622	Yes		22	0.39	17	0
chr16	32009885	32108583	Yes		14	0.32	9	0
chr16	32117983	32284829	Yes		20	0.15	6	2
chr16	32739755	33358391	Yes		22	0.57	25	4
chr16	33491352	33837385	Yes		22	0.07	3	2
chr16	33884906	33984200	Yes		15	0.07	2	0
chr16	70122074	70177522	Yes		14	0.57	16	5
chr16	75206301	75222802	No		4	0.5	4	40
chr17	16754850	16845985	Yes		23	0.17	8	8
chr17	18409166	18503176	Yes		19	0.18	7	11
chr17	21304384	21351378	No		31	0.35	22	0

chr17	21399734	21448998	No		32	0.36	23	0
chr17	26935981	27009054	No		31	0.34	21	0
chr17	37914195	37983238	Yes		11	0.27	6	1
chr17	45584409	46295299	Yes		32	0.23	15	2
chr19	24330850	24410734	No		24	0.31	15	0
chr19	27339729	27389526	No		24	0.08	4	0
chr20	25842796	26011703	Yes		28	0.3	17	7
chr20	26069364	26338933	Yes		29	0.07	4	4
chr21	9887935	10169868	Yes		28	0.27	15	0
chr21	10510681	10740368	Yes		28	0.36	20	4
chr21	12965809	13083741	Close to/including gaps	Close to the gap chr21:12915808-12965808	27	0.26	14	0
chr21	13982706	14066821	Yes		25	0.06	3	2
chr22	15543181	15927980	Yes		26	0.62	32	2
chr22	16390097	16572245	Yes		26	0.1	5	0
chr22	18726137	18885045	Yes		20	0.45	18	3
chr22	18511378	18659564	Yes		4	0.38	3	3
chr22	21109554	21421587	Yes		19	0.34	13	18
chr22	21438720	21457842	Yes		3	0.5	3	10
chrX	36608469	37080183	Close to/including gaps	Close to the gap chrX:37099262-37285837	30	0.06	4	0
chrX	49163588	49264019	Close to/including gaps	Close to the gap chrX:49348394-49528394	23	0.06	3	8
chrX	52071920	52177020	Yes		17	0.37	13	0
chrX	63252842	63289305	No		9	0	0	2
chrX	103988035	104050515	Yes		17	0.83	28	28
chrX	120087061	120150566	Yes		13	0.29	8	8
chrX	135157653	135214201	Yes		13	0.48	12	6
chrX	141108461	141474368	Yes		29	0.12	7	3
chrX	149682830	149720814	Yes		9	0.75	14	0
chrX	153149489	153249413	Yes		14	0.33	9	14
chrY	21567242	21739542	Yes		21	0.1	4	0
chrY	22209549	22379273	Yes		17	0.12	4	0

Supplementary References

1. Wolfe, D., Dudek, S., Ritchie, M. D. & Pendergrass, S. A. Visualizing genomic information across chromosomes with PhenoGram. *BioData Min.* **6,** 18 (2013).
2. Sudmant, P. H. *et al.* An integrated map of structural variation in 2,504 human genomes. *Nature* **526,** 75–81 (2015).
3. Mallick, S. *et al.* The Simons Genome Diversity Project: 300 genomes from 142 diverse populations. *Nature* **538,** 201–206 (2016).
4. Telenti, A. *et al.* Deep sequencing of 10,000 human genomes. *Proc. Natl. Acad. Sci. U. S. A.* **113,** 11901–11906 (2016).
5. 1000 Genomes Project Consortium *et al.* A global reference for human genetic variation. *Nature* **526,** 68–74 (2015).
6. Weisenfeld, N. I., Kumar, V., Shah, P., Church, D. M. & Jaffe, D. B. Direct determination of diploid genome sequences. *Genome Res.* **27,** 757–767 (2017).
7. Kurtz, S. *et al.* Versatile and open software for comparing large genomes. *Genome Biol.* **5,** R12 (2004).
8. Patterson, N., Price, A. L. & Reich, D. Population Structure and Eigenanalysis. *PLoS Genet.* **2,** e190 (2006).
9. Kirk, D. Computer-based saturation curve analysis. *The Shot Peener* 24–30 (2006).
10. Jee, J. *et al.* ACT: Aggregation and correlation toolbox for analyses of genome tracks. *Bioinformatics* **27,** 1152–1154 (2011).
11. Sanders, A. D. *et al.* Characterizing polymorphic inversions in human genomes by single-cell sequencing. *Genome Res.* **26,** 1575–1587 (2016).
12. Poznik, G. D. *et al.* Sequencing Y Chromosomes Resolves Discrepancy in Time to Common Ancestor of Males Versus Females. *Science (80-.).* **341,** 562–565 (2013).
13. Tyner, C. *et al.* The UCSC Genome Browser database: 2017 update. *Nucleic Acids*

Res. **45,** D626–D634 (2017).

14. Leung, A. K.-Y., Jin, N., Yip, K. Y. & Chan, T.-F. OMTools: a software package for visualizing and processing optical mapping data. *Bioinformatics* **33,** 2933–2935 (2017).
15. Quinlan, A. R. & Hall, I. M. BEDTools: a flexible suite of utilities for comparing genomic features. *Bioinformatics* **26,** 841–842 (2010).
16. Harris, R. S. Improved Pairwise Alignment of Genomic DNA. (Pennsylvania State University, 2007).
17. Smit, A., Hubley, R. & P, G. RepeatMasker Open-4.0.
18. Nattestad, M. & Schatz, M. C. Assemblytics: a web analytics tool for the detection of variants from an assembly. *Bioinformatics* **32,** 3021–3023 (2016).
19. Schrider, D. R. *et al.* Gene copy-number polymorphism caused by retrotransposition in humans. *PLoS Genet.* **9,** e1003242 (2013).
20. Langmead, B. & Salzberg, S. L. Fast gapped-read alignment with Bowtie 2. *Nat. Methods* **9,** 357 (2012).
21. Rice, P., Longden, I. & Bleasby, A. EMBOSS: the European molecular biology open software suite. *Trends Genet.* **16,** 276–277 (2000).
22. Altschul, S. F., Gish, W., Miller, W., Myers, E. W. & Lipman, D. J. Basic local alignment search tool. *J. Mol. Biol.* **215,** 403–410 (1990).
