# Supplementary material for: Genome maps across 26 human populations reveal population-specific patterns of structural variation
Source: Nat Commun. 2019 Mar 4;10:1025. doi: 10.1038/s41467-019-08992-7 (PMC6399254; doi:10.1038/s41467-019-08992-7)
Supplement: Supplementary file 3 — Description of Additional Supplementary Files [file 41467_2019_8992_MOESM3_ESM.pdf]

Description of Additional Supplementary Files

Supplemental Data 1. Summary de novo assembly statistics of individual samples.
Supplementary Data 2. Regions of the reference genome that are inaccessible with genome mapping.
Supplementary Data 3. Coordinates and annotation of regions with complex structural variations.
Supplementary Data 4. Large insertions and deletions found in individual samples.
Supplementary Data 5. The list of inversions found in samples.
Supplementary Data 6. The list of multiple indels found in samples.
Supplementary Data 7. The list of translocation breakends.
Supplementary Data 8. The list of duplications and other structural variations.
Supplementary Data 9. The list of indels with statistically significant size differences among super-populations.
Supplementary Data 10. List of copy number variations analyzed by the multiple alignment approach.
Supplementary Data 11. Reference gaps closed with 10X data.

Response to requests
(Requests in *italics*, our response in blue)

EDITORIAL REQUESTS:
We would specifically ask that you include a more thoughtful consideration of how the newly released data can be used by the community, as per Reviewer #3's request.
Done. See new sentences in the Discussion Section.

At the same time we ask that you edit your manuscript to comply with our format and style requirements and to maximise the accessibility and therefore the impact of your work.
Done.

TITLE PAGE
** Our format allows only two specific author tagging statements, "These authors contributed equally" and "These authors jointly supervised this work", with one of each tag allowed.*
Done.

** Please ensure that all affiliations are in the correct sequential order according to their position in the author list. Affiliation 1 must be associated with the first author. Please see this article for further detail: https://www.nature.com/articles/s41467-018-04254-0.pdf*
Done.

** Please shorten the abstract to 150 words or fewer.*
Done.

** When discussing the current work in the abstract, please use the present tense.*
Done.

MAIN TEXT
** Please shorten the main manuscript text (Introduction, Results, and Discussion, not including figure legends or Methods) to approximately 5,000 words or fewer.*

** Please use the present tense when discussing the current work in the Introduction.*
Done.

** Please shorten all subheadings in the Results section to fewer than 60 characters including spaces.*
Done.

LANGUAGE AND STYLE
** Please avoid using speech marks around words or phrases. In most cases they are unnecessary.*
Done.

** Please make sure that mathematical terms throughout your manuscript and Supplementary Information (including in figures, figure axes, and legends) conform strictly to the following guidelines. Equations should be supplied in editable format, and not as images. Scalar variables (e.g. x, V, χ) should be typeset in italic, whereas multi-letter*

variables should be formatted in roman. Constants (e.g. ħ, G, c) should be typeset in italics (the only exceptions being e, i, π, which should be typeset in Roman) and vectors (such as r, the wavevector k, or the magnetic field vector B) should be typeset in bold without italics. In contrast, subscripts and superscripts should only be italicised if they too are variables or constants. Those that are labels (such as the 'c' in the critical temperature, T_c, the 'F' in the Fermi energy, E_F, or the 'crit' in the critical current, I_crit) should be typeset in roman. To avoid doubt, unit dimensions should be expressed using negative integers (e.g. kg m^-1 s^-2, not kg/ms^2) or the word 'per'.
Done.

* Wherever p-values are stated in the text and figure legends, please also state the name of the statistical test.
Done.

METHODS AND DATA
* Please provide a full Methods section in the main manuscript file. Please note that there are no word limits to the Methods section. The Methods section must include subheadings of fewer than 60 characters including spaces. Please do not number the headings.
Done.

* Please confirm that you have complied with all relevant ethical regulations for work with human participants, and that informed consent was obtained. Please state this in the Methods section, including the name of the board and institution that approved the study protocol.
No human subjects involved. All samples are from the NIH collection in a public repository maintained by the Coriell Institute.

* All Nature Communications manuscripts must include a section titled "Data Availability" as a separate section after the Methods section and before the References. For more information on this policy, and a list of examples, please see http://www.nature.com/authors/policies/data/data-availability-statements-data-citations.pdf
Done.

* DATA SOURCES: We strongly encourage authors to deposit all new data associated with the paper in a persistent repository where they can be freely and enduringly accessed. We recommend submitting the data to discipline-specific, community-recognized repositories, where possible and a list of recommended repositories is provided here: http://www.nature.com/sdata/policies/repositories
If a community resource is unavailable, data can be submitted to generalist repositories such as figshare (https://figshare.com/) or Dryad Digital Repository (https://protect2.fireeye.com/url?k=cdf71b81-91b776d7-cdf73c9c-0cc47adb57f0-5b00d7f8899114f7&u=http://datadryad.org/). Please provide a unique identifier for the data (for example a DOI or a permanent URL) in the "Data Availability" section, if possible. If the repository does not provide identifiers, we encourage authors to supply the search terms that will return the data. For data that have been obtained from publically available sources, please provide a URL and the specific data product name in the "Data Availability" section. Data with a DOI should be included in the reference list and cited where relevant.
Please refer to our data policies

here: http://www.nature.com/authors/policies/availability.html
Done.

** Please ensure that all novel nucleotide sequences are deposited in the NCBI Genbank nucleotide database, and that accession codes are provided in the "Data Availability" section.*
No novel nucleotide sequences reported in our study.

** To ensure correct hyperlinking of the accession codes in your manuscript, please add the hyperlink or DOI in square brackets directly after the code throughout (for example, "5XRN [http://dx.doi.org/10.2210/pdb5XRN/pdb]", "1483958 [https://dx.doi.org/10.5517/ccdc.csd.cc1lt5m6]", "SRP109982 [https://www.ncbi.nlm.nih.gov/sra/?term=SRP109982]" or "NQLW00000000 [https://www.ncbi.nlm.nih.gov/assembly/GCA_002312845.1/]").*
Done.

END NOTES
** Please provide a "Competing Interests" section after the "Author Contributions" section that refers to all authors. If there are no competing interests, please add the statement "The authors declare no competing interests."*
Done.

** Please ensure the references are in the standard Nature format and follow the sequence: author list, title of paper, name of journal, volume number, initial-final page numbers or article number (year). Please note that dois are required only for online-only publications and correct journal abbreviations should be given.*
Done.

** Please update any preprints in the reference list with details of the published paper, if possible. If the paper has not been published, please cite the preprint in the style: Babichev, S. A., Ries, J. & Lvovsky, A. I. Quantum scissors: teleportation of single-mode optical states by means of a nonlocal single photon. Preprint at http://arXiv.org/quant-ph/0208066 (2002).*
None cited.

** Please update any incomplete web links for DOI references and preprints.*
Not applicable.

DISPLAY ITEMS
** Please check whether your manuscript or Supplementary Information contain third-party images, such as figures from the literature, stock photos, clip art or commercial satellite and map data. We strongly discourage the use or adaptation of previously published images, but if this is unavoidable, please request the necessary rights documentation to re-use such material from the relevant copyright holders and return this to us when you submit your revised manuscript.*
Not applicable.

** Please ensure that figure legend titles are brief - they should not occupy more than one line in the final proof.*
In compliance.

** Please ensure that all elements of boxplots are defined in the associated figure legend (centre line, bounds of box and whiskers).*
Done.

** Please ensure that the corresponding dot plots are overlaid in the bar charts.*
Done.

** Data in tables must be free from bold/italic formatting unless this has been clearly defined in the footnote. Tables need to be black and white, fit onto a single A4 portrait page and can contain only one row of column titles. Finally, we are unable to merge cells or include vertical dividing lines or diagonal lines. Please format your tables accordingly.*
In compliance.

** In each figure and supplementary figure where error bars are used, they must be defined. One statement at the end of each figure is sufficient if the error bars are equivalent throughout the figure.*
Done.

SUPPLEMENTARY INFORMATION
** We do not edit Supplementary Information (SI) files; they will be uploaded with the published article as they are submitted with the final version of your manuscript. Any tracked changes should be removed from the file.*
Done.

** The only section headings permitted in the Supplementary Information are Supplementary Figures, Supplementary Tables, Supplementary Methods, Supplementary Notes, Supplementary Discussion, Supplementary References. All other section headings and numbering should be removed or relabelled.*
Done.

** In the Supplementary Information file and the main manuscript text, supplementary items must be labelled and cited using only the following formats: Supplementary Figure 1, Supplementary Table 1, Supplementary Methods, Supplementary Note 1, Supplementary Discussion, and Supplementary References. Please note the use of "Supplementary" and that we do not use the "S" prefix.*
Done.

** Please merge all Supplementary Information files into one PDF document. Only Supplementary Movie/Audio/Data/Software files should be submitted separately from the Supplementary Information.*
Done.

** Please label supplementary equations sequentially as (1), (2), (3), etc. (and without an "S" prefix) (if applicable).*
Done.

** Supplementary References should appear at the end of the Supplementary Information file, and should be self-contained and numbered from 1. References mentioned in both the main text and the Supplementary Information should be part of both reference lists so that*

the Supplementary Information does not refer to the reference list in the main paper and vice versa.
Done.

** Large datasets and unwieldy tables should be supplied as Supplementary Data files, whereas smaller tables should be supplied as supplementary tables. As such, please include Supplementary Tables 3, 7, 8, 11, 12, 14, 15, 16a, 16b as Supplementary Tables in the merged SI pdf file. All other (large) Supplementary Tables should be submitted as "Supplementary Data" instead (i.e. 1, 2, 4, 5, 6a-d, 9, 10, 13), as individual excel files (one file per dataset). Please also note that we do not allow panels (a, b, c etc) for Tables. Please renumber all Supplementary items accordingly and also change the callout in the article.*
Done.

** Please supply legends for each Supplementary Movie/Audio/Data file in your cover letter (not in the Supplementary Information file). Please label each files as Supplementary Movie/Audio/Data 1, etc.*
Not applicable.

** Your paper will be accompanied by a two-sentence Editor's summary, of between 250-300 characters including spaces, when it is published on our homepage. Could you please approve the draft summary below or provide us with a suitably edited version.*

"Large structural variants (SV) are understudied in human genetics research because of the difficulty to detect them in the routinely generated short-read sequencing data. Here, the authors generate optical genome maps of 154 individuals from 26 populations that allow comprehensive examination of large SVs."
The summary reads well and to the point. Approved.

** As part of our efforts to communicate our content to a wider audience, we endeavour to highlight papers published in Nature Communications on the journal's Twitter account (@NatureComms). If you would like us to mention authors, institutions or lab groups in these tweets, please provide the relevant twitter handles in your cover letter upon resubmission.*
None.

** If you opted into the journal hosting details of a preprint version of your manuscript via a link on our dedicated website (https://nature-research-under-consideration.nature.com), it will remain on this site while you are revising your manuscript, as we consider the file to remain active. Should you wish to remove these details, please emailnaturecommunications@nature.com indicating your manuscript number and the link on our website that was previously sent to you. Please see our pre-publicity policy athttp://www.nature.com/authors/policies/confidentiality.html For more information, please refer to our FAQ page at https://nature-research-under-consideration.nature.com/posts/19641-frequently-asked-questions*
Not opting into the preprint hosting offer.

** In recognition of the time and expertise our reviewers provide to Nature Communications's editorial process, as of November, 2018, we formally acknowledge their contribution to the external peer review of articles published in the journal. All peer-*

reviewed content will carry an anonymous statement of peer reviewer acknowledgement, and for those reviewers who give their consent, we will publish their names alongside the published article. For more information, please refer to our FAQ page at https://www.nature.com/documents/ncomms-reviewer-information.pdf
Sounds good.

OPEN ACCESS:
Nature Communications is a fully open access journal. Articles are made freely accessible on publication under a CC BY license (Creative Commons Attribution 4.0 International License). This license allows maximum dissemination and re-use of open access materials and is preferred by many research funding bodies.
Noted.

SUBMISSION INFORMATION:
In order to accept your paper, we require the following:
** A cover letter describing your response to our editorial requests.*
Done.
